# Supplementary material for: Merle phenotypes in dogs – SILV SINE insertions from Mc to Mh
Source: PLoS One. 2018 Sep 20;13(9):e0198536. doi: 10.1371/journal.pone.0198536 (PMC6147463; doi:10.1371/journal.pone.0198536)
Supplement: S1 Table — ASD–Australian Shepherd Dog, AK–Australian Koolie, BC–Border Collie, D–Dachshund, FB–French Bulldog, LC–Louisiana Catahoula, LD–Labradoodle, MAS–Miniature American Shepherd, MAUS–Miniature Australian Shepherd, PS–Pyrenean Shepherd, RC–Rough Collie, SSD–Shetland Sheepdog, WS–Welsh Sheepdog, n–number of tested dogs. The size of the wild type allele is 171 bp. (DOCX) [file pone.0198536.s001.docx]

| **BREED** | **n** | **Mc** | **Mc+** | **Ma** | **Ma+** | **M** | **Mh** |
| --- | --- | --- | --- | --- | --- | --- | --- |
| **LC** | 73 | 208–230 | 233–245 | 247–253 | 255–264 | 265–268 | 271–277 |
| **ASD** | 40 | 216–230 | 233–243 | 252–254 | 259–259 | 266–268 | 269–274 |
| **AK** | 23 | 213–230 | 231–235 |  | 262–264 | 265–268 | 269–278 |
| **BC** | 18 | 222–225 | 246 | 254 | 256–262 | 268 | 269–274 |
| **D** | 9 |  |  |  | 258–260 | 268 | 274 |
| **RC** | 3 | 211 |  |  | 260 | 267 |  |
| **WS** | 3 |  | 231 |  |  | 268 | 272–273 |
| **MAUS** | 2 | 217 |  |  | 262 |  | 269–273 |
| **MAS** | 2 |  |  |  |  | 267 |  |
| **FB** | 2 |  |  |  |  | 265 |  |
| **SSD** | 2 | 220–223 |  |  |  |  | 271 |
| **M** | 2 | 226 |  |  |  | 267 |  |
| **PS** | 1 |  |  |  |  | 265 |  |
| **LD** | 1 |  |  |  |  | 266 |  |
| **Average range of Merle alleles in bp** |  | 208–230 | 231–245 | 247–254 | 255–264 | 265–269 | 269–277 |
