# Supplementary material for: Merle phenotypes in dogs – SILV SINE insertions from Mc to Mh
Source: PLoS One. 2018 Sep 20;13(9):e0198536. doi: 10.1371/journal.pone.0198536 (PMC6147463; doi:10.1371/journal.pone.0198536)
Supplement: S2 Table — The table gives details on the length of the individual Merle alleles in mosaic dogs. (DOCX) [file pone.0198536.s002.docx]

| **BREED** | **SAMPLE CODE** | **MERLE ALLELIC STATUS** | **MAJOR ALLELE/S** | **MINOR ALLELE/S** | **SHORTENED MERLE ALLELES** |
| --- | --- | --- | --- | --- | --- |
| **Catahoula** | AE512 | [Mc]/[Mc+]/Ma/Mh  [218]/[238]/250/277 | 250/277 | 218/238 | Mh and/or Ma to Mc/Mc+ |
| **Australian Koolie** | AF521 | m/[Mc]/Mh  m/[225]/269 | 269 | 225 | Mh to Mc |
| **Australian Shepherd** | AF509 | [Mc]/[Mc]/ Mh/Mh  [220]/[230] /269/269 | 269/269 | 220/230 | Mh to Mc |
| **Border Collie** | AF459 | m/[Mc]/Mh  m/[225]/271 | 271 | 225 | Mh to Mc |
| **Australian Shepherd** | AE877 | m/[Mc]/Mh  m/[230]/270 | 270 | 230 | Mh to Mc |
| **Australian Koolie** | AF508 | [Mc]/[Mc]/[Mc]/[Mc+]/Mh/Mh [213]/[220]/[230]/[235]/269/269 | 269/269 | 213/220/  230/235 | Mh to Mc and/or Mc+ |
| **Shetland Sheepdog** | AE982 | m/[Mc]/Mh  m/[220]/271 | 271 | 220 | Mh to Mc |
| **Australian Koolie** | AF513 | m/[Mc+]/Mh  m/[231]/278 | 278 | 231 | Mh to Mc+ |
| **Australian Shepherd** | AF074 | m/[Ma]/Mh  m/[254]/270 | 270 | 254 | Mh to Ma |
| **Miniature Australian Shepherd** | AE820 | m/[Ma+]/Mh  m/[262]/273 | 273 | 262 | Mh to Ma+ |
| **Catahoula** | AE870 | m/[Mc]/M  m/[208]/265 | 265 | 208 | M to Mc |
| **French Bulldog** | AE941 | m/[Mc+]/M  m/[241]/265 | 265 | 241 | M to Mc+ |
| **Catahoula** | AE787 | [Ma]/M/M  [251]/265/265 | 265/265 | 251 | M to Ma |
| **Catahoula** | AF132 | m/[Ma+]/M  m/[257]/266 | 266 | 257 | M to Ma+ |
| **Border Collie** | AF163 | m/[Ma+]/M  m/[260]/268 | 268 | 260 | M to Ma+ |
| **Dachshund** | AF020 | m/[Ma+]/M  m/[258]/268 | 268 | 258 | M to Ma+ |
| **Border Collie** | AF163 | m/[Ma+]/M  m/[260]/268 | 268 | 260 | M to Ma+ |
| **Catahoula** | AE956 | [Mc]/Ma/M  [225]/251/265 | 251/265 | 225 | M and/or Ma to Mc |
| **Catahoula** | AE515 | [Mc]/Ma/M  [221]/252/267 | 252/267 | 221 | M or Ma to Mc |
| **Border Collie** | AF514 | m/[Mc]/[Mc+]/Ma+ m/[222]/[246]/262 | 262 | 222/246 | Ma+ to Mc+ and/or Mc |
| **Australian Koolie** | AF225 | m/[Mc]/Ma+  m/[215]/262 | 262 | 215 | Ma+ to Mc |
| **Australian Shepherd** | AE786 | m/[Mc+]/Ma  m/[243]/252 | 252 | 243 | Ma to Mc+ |
| **Catahoula** | AF265 | m/[Mc]/Mc+  m/[222]/239 | 239 | 222 | Mc+ to Mc |
| **Wesh Sheepdog** | AF613 | m/[Mc+]/Mh  m/[231]/273 | 273 | 231 | Mh to Mc+ |
| **Australian Shepherd** | AF652 | m/[Mc]/M  m/[226]/266 | 266 | 226 | M to Mc |
| **Border Collie** | AF781 | m/[Ma+]/M  m/[261]/267 | 267 | 261 | M to Ma+ |
| **Border Collie** | AF782 | m [Ma+]/Mh  m [257] 270 | 270 | 257 | Mh to Ma+ |
| **Mudi** | AF650 | m/[Mc]/M  m/[226]/267 | 267 | 226 | M to Mc |
