# Supplementary material for: Merle phenotypes in dogs – SILV SINE insertions from Mc to Mh
Source: PLoS One. 2018 Sep 20;13(9):e0198536. doi: 10.1371/journal.pone.0198536 (PMC6147463; doi:10.1371/journal.pone.0198536)
Supplement: S3 Table — The table gives details on pedigree relationships of dogs involved in the study. (DOCX) [file pone.0198536.s003.docx]

| **SIRE** | **DAM** | **OFFSPRING** | **BREED** |
| --- | --- | --- | --- |
| AF016 | AF017 | AF022, AF019, AF020, AF018 | Dachshund |
| AF014 | AF013 | AF008, AF009, AF010, AF011, AF012, AF015, AF237 | Australian Shepherd |
| AF014 | N/A | AF239, AF241 | Australian Shepherd |
| AE939 | AE869 | AE803, AE903 | Australian Shepherd |
| N/A | N/A | AE868, AE869 | Australian Shepherd |
| N/A | AE877 | AF050 | Australian Shepherd |
| AF761 | AF762 | AF758, AF759, AF760 | Australian Shepherd |
| N/A | N/A | AE572, AF146 | Catahoula |
| AE574 | AF146 | AF161 | Catahoula |
| AE515 | AF515 | AF612 | Catahoula |
| N/A | N/A | AE428, AE573, AF341 | Catahoula |
| N/A | AE904 | AE512 | Catahoula |
| N/A | N/A | AF699, AF352 | Catahoula |
| AF132 | AF053 | AE878, AE804, AF399 | Catahoula |
| AE505 | AF402 | AE504, AF231 | Catahoula |
| AE505 | N/A | AF101 | Catahoula |
| AF099 | AE622 | AF232 | Catahoula |
| AF129 | AE787 | AE740, AE515 | Catahoula |
| AF133 | N/A | AF176 | Catahoula |
| N/A | N/A | AE463, AE535 | Catahoula |
| AE956 | N/A | AF185 | Catahoula |
| AE505 | AE852 | AF196 | Catahoula |
| AF380 | AF038 | AF379, AF378 | Catahoula |
| AF330 | AF510 | AF333, AF335 | Catahoula |
| AF333 | AF510 | AF334 | Catahoula |
| AF333 | AF331 | AF332 | Catahoula |
| AF513 | N/A | AF400 | Australian Koolie |
| AF508 | AF484 | AF327, AF521, AF485 | Australian Koolie |
| N/A | AE819 | AE818 | Border Collie |
| AF056 | N/A | AF514 | Border Collie |
| N/A | N/A | AF649, AF650 | Mudi |
