# Supplementary material for: Merle phenotypes in dogs – SILV SINE insertions from Mc to Mh
Source: PLoS One. 2018 Sep 20;13(9):e0198536. doi: 10.1371/journal.pone.0198536 (PMC6147463; doi:10.1371/journal.pone.0198536)
Supplement: S4 Table — The table shows the details of Merle allelic mosaicism in 30 individuals identified in our study. (DOCX) [file pone.0198536.s004.docx]

| **Sample code** | **Allelic status of Merle alleles** | **Size of Merle alleles in bp** | **Breed** | **Biological material** | **SuperColorLocus reference**  **(Table S5)** |
| --- | --- | --- | --- | --- | --- |
| AE512 | [Mc]/[Mc+]/Ma/Mh | [218]/[238]/250/277 | Catahoula | buccal swab |  |
| AE956 | [Mc]/Ma/M | [225]/251/265 | Catahoula | buccal swab |  |
| AE787 | [Ma]/M/M | [251]/265/265 | Catahoula | buccal swab |  |
| AE515 | [Mc]/Ma/M | [221]/252/267 | Catahoula | peripheral blood |  |
| AE786 | m/[Mc+]/Ma | m/[243]/252 | Australian Shepherd | hair |  |
| AE820 | m/[Ma+]/Mh | m/[262]/273 | Miniature Australian Shepherd | peripheral blood |  |
| AE982 | m/[Mc]/Mh | m/[220]/271 | Shetland Sheepdog | buccal swab |  |
| AF074 | m/[Ma]/Mh | m/[254]/270 | Australian Shepherd | buccal swab |  |
| AF104 | m/[Mc+]/M | m/[241]/265 | French Bulldog | hair |  |
| AF163 | m/[Ma+]/M | m/[260]/268 | Border Collie | buccal swab |  |
| AF038 | m/Ma+/[M] | m/258/[266] | Catahoula | buccal swab |  |
| AF508 | [Mc]/[Mc]/[Mc]/Mc+]/Mh/Mh | [213]/[220]/[230]/[235]/269/269 | Australian Koolie | semen | AF174 - buccal swab |
| AF225 | m/[Mc]/Ma+ | m/[215]/262 | Australian Koolie | buccal swab |  |
| AE132 | m/[Ma+]/M | m/[257]/266 | Catahoula | hair | AE802 - buccal swab |
| AE870 | m/[Mc]/M | m/[208]/265 | Catahoula | peripheral blood |  |
| AF265 | m/[Mc]/Mc+ | m/[222]/239 | Catahoula | hair |  |
| AF020 | m/[Ma+]/M | m/[258]/268 | Dachshund | buccal swab |  |
| AE943 | m/Mc+/[M] | m/233/[267] | Australian Shepherd | semen |  |
| AE877 | m/[Mc]/Mh | m/[230]/270 | Australian Shepherd | buccal swab |  |
| AF273 | m/Ma/[M] | m/250/[266] | Catahoula | peripheral blood |  |
| AF459 | m/[Mc]/Mh | m/[225]/271 | Border Collie | buccal swab |  |
| AF513 | m/[Mc+]/Mh | m/[231]/278 | Australian Koolie | hair |  |
| AF514 | m/[Mc]/[Mc+]/Ma+ | m/[222]/[246]/262 | Border Collie | buccal swab |  |
| AF509 | [Mc]/[Mc]/ Mh/Mh | [220]/[230] /269 269 | Australian Shepherd | hair |  |
| AF521 | m/[Mc]/Mh | m/[225]/269 | Australian Koolie | buccal swab |  |
| AF613 | m/[Mc+]/Mh | m/[231]/273 | Welsh Sheepdog | hair |  |
| AF652 | m/[Mc/ M | m/[226]/266 | Australian Shepherd | hair |  |
| AF781 | m/[Ma+]/M | m/[261]/267 | Border Collie | buccal swab |  |
| AF782 | m/[Ma+]/Mh | m/[257]/270 | Border Collie | buccal swab |  |
| AF650 | m/[Mc]/M | m/[226]/267 | Mudi | buccal swab |  |
