# Supplementary material for: Merle phenotypes in dogs – SILV SINE insertions from Mc to Mh
Source: PLoS One. 2018 Sep 20;13(9):e0198536. doi: 10.1371/journal.pone.0198536 (PMC6147463; doi:10.1371/journal.pone.0198536)
Supplement: S5 Table — shows the genotype summary of the so far recognized coat color loci in dog and their modifiers relevant for the study; all photographs of the genotyped animals can be found in Figures A-X in S1 Fig, respectively. (DOCX) [file pone.0198536.s005.docx]

| **SuperColorLocus – GENOTYPE SUMMARY** | | | | | | | | | | | | | |
| --- | --- | --- | --- | --- | --- | --- | --- | --- | --- | --- | --- | --- | --- |
| **Sample code** | **Breed** | **Locus E (Alleles E, e)** | **Locus E (Allele Em)** | **Locus E (allele Eg)** | **Locus E (allele Eh)** | **Locus K (alleles K, k*)** | **Locus A (alleles Ay, aw, at, a)** | **Locus B (allele bc)** | **Locus B (allele bs)** | **Locus B (allele bd)** | **Locus D** | **Locus S** | **Locus H (Harlequin - PSMB7)** |
| **AE428** | **Catahoula** | E/E | wt/wt | wt/wt | wt/wt | K/k* | at/at | B/B | B/bs | B/B | D/D | S/S | h/h |
| **AE463** | **Catahoula** | E/E | Em/Em | wt/wt | wt/wt | K/k* | at/at | B/B | B/B | B/B | D/d | S/S | h/h |
| **AE479** | **Catahoula** | E/e | wt/wt | wt/wt | wt/wt | K/k* | at/at | B/B | B/B | B/B | D/D | S/S | h/h |
| **AE487** | **Catahoula** | E/E | Em/wt | wt/wt | wt/wt | K/k* | Ay/aw | B/B | B/B | B/B | D/D | S/S | h/h |
| **AE488** | **Catahoula** | E/E | wt/wt | wt/wt | wt/wt | K/k* | at/at | B/B | B/bs | B/B | D/d | S/sp | h/h |
| **AE504** | **Catahoula** | E/e | wt/wt | wt/wt | wt/wt | k*/k* | at/a | B/B | B/bs | B/B | D/D | S/S | h/h |
| **AE505** | **Catahoula** | E/E | Em/wt | wt/wt | wt/wt | k*/k* | at/at | B/B | B/B | B/B | D/D | S/S | h/h |
| **AE512** | **Catahoula** | E/E | Em/Em | wt/wt | wt/wt | K/k* | at/a | B/B | B/B | B/B | D/D | S/S | h/h |
| **AE513** | **Catahoula** | E/E | wt/wt | wt/wt | wt/wt | k*/k* | at/at | B/B | B/B | B/B | D/D | S/S | h/h |
| **AE514** | **Catahoula** | E/E | wt/wt | wt/wt | wt/wt | k*/k* | at/at | B/B | bs/bs | B/B | D/d | S/S | h/h |
| **AE515** | **Catahoula** | E/E | Em/wt | wt/wt | wt/wt | K/k* | at/at | B/B | bs/bs | B/B | D/D | S/sp | h/h |
| **AE535** | **Catahoula** | E/E | Em/Em | wt/wt | wt/wt | K/k* | at/at | B/B | B/B | B/B | D/D | S/S | h/h |
| **AE572** | **Catahoula** | E/E | wt/wt | wt/wt | wt/wt | k*/k* | at/at | B/B | bs/bs | B/B | D/D | S/S | h/h |
| **AE573** | **Catahoula** | E/E | wt/wt | wt/wt | wt/wt | K/k* | at/at | B/B | B/B | B/B | D/d | S/S | h/h |
| **AE574** | **Catahoula** | E/E | Em/wt | wt/wt | wt/wt | K/k* | at/at | B/bc | B/bs | B/bd | D/d | S/S | h/h |
| **AE612** | **Catahoula** | E/E | Em/wt | wt/wt | wt/wt | K/k* | at/a | B/B | B/bs | B/B | D/D | S/S | h/h |
| **AE621** | **Catahoula** | E/E | Em/Em | wt/wt | wt/wt | K/k* | at/at | B/B | B/bs | B/B | D/D | S/S | h/h |
| **AE622** | **Catahoula** | E/E | Em/wt | wt/wt | wt/wt | K/k* | at/at | B/B | B/bs | B/B | D/D | S/S | h/h |
| **AE693** | **Catahoula** | E/E | wt/wt | wt/wt | wt/wt | K/k* | at/at | B/B | bs/bs | B/B | D/d | sp/sp | h/h |
| **AE740** | **Catahoula** | E/E | Em/Em | wt/wt | wt/wt | K/k* | at/at | B/B | bs/bs | B/B | D/d | S/S | h/h |
| **AE786** | **Australian Shepherd** | E/E | Em/wt | wt/wt | wt/wt | k*/k* | at/at | B/B | B/bs | B/B | D/D | S/S | h/h |
| **AE787** | **Catahoula** | E/E | Em/wt | wt/wt | wt/wt | K/k* | at/at | B/bc | B/bs | B/bd | D/D | S/sp | h/h |
| **AE802** | **Catahoula** | E/E | Em/Em | wt/wt | wt/wt | k*/k* | at/at | B/B | bs/bs | B/B | D/D | S/sp | h/h |
| **AE803** | **Australian Shepherd** | E/E | wt/wt | wt/wt | wt/wt | k*/k* | at/at | B/B | B/bs | B/B | D/D | S/S | h/h |
| **AE804** | **Catahoula** | E/E | Em/wt | wt/wt | wt/wt | K/k* | at/at | B/B | B/bs | B/B | D/D | S/sp | h/h |
| **AE817** | **Border Collie** | E/e | Em/wt | wt/wt | wt/wt | K/K | aw/at | B/B | B/B | B/B | D/d | S/S | h/h |
| **AE818** | **Border Collie** | E/E | Em/wt | wt/wt | wt/wt | K/k* | at/at | B/B | B/bs | B/B | D/d | S/sp | h/h |
| **AE819** | **Border Collie** | E/E | Em/Em | wt/wt | wt/wt | K/k* | at/at | B/B | B/B | B/B | D/d | S/S | h/h |
| **AE820** | **Miniature Australian Shepherd** | E/E | wt/wt | wt/wt | wt/wt | k*/k* | at/a | B/B | B/B | B/B | D/D | S/S | h/h |
| **AE845** | **Miniature American Shepherd** | E/e | wt/wt | wt/wt | wt/wt | k*/k* | at/a | B/bc | B/bs | B/bd | D/D | S/S | h/h |
| **AE846** | **Miniature American Shepherd** | E/E | wt/wt | wt/wt | wt/wt | k*/k* | at/at | B/B | B/bs | B/B | D/D | S/S | h/h |
| **AE852** | **Catahoula** | E/E | Em/Em | wt/wt | wt/wt | K/k* | at/a | B/B | B/bs | B/B | D/D | S/S | h/h |
| **AE868** | **Australian Shepherd** | E/E | wt/wt | wt/wt | wt/wt | k*/k* | at/at | B/B | bs/bs | B/B | D/D | S/S | h/h |
| **AE869** | **Australian Shepherd** | E/E | wt/wt | wt/wt | wt/wt | k*/k* | at/at | B/B | B/bs | B/B | D/D | S/S | h/h |
| **AE870** | **Catahoula** | E/E | Em/wt | wt/wt | wt/wt | K/k* | at/a | B/B | bs/bs | B/B | D/D | S/S | h/h |
| **AE877** | **Australian Shepherd** | E/E | wt/wt | wt/wt | wt/wt | k*/k* | at/at | B/B | B/bs | B/B | D/D | S/S | h/h |
| **AE878** | **Catahoula** | E/E | Em/wt | wt/wt | wt/wt | K/k* | at/at | B/B | bs/bs | B/B | D/D | S/S | h/h |
| **AE903** | **Australian Shepherd** | E/E | wt/wt | wt/wt | wt/wt | k*/k* | at/at | B/B | bs/bs | B/B | D/D | S/S | h/h |
| **AE904** | **Catahoula** | E/E | Em/Em | wt/wt | wt/wt | K/k* | at/a | B/B | B/bs | B/B | D/D | S/S | h/h |
| **AE905** | **Catahoula** | E/E | Em/wt | wt/wt | wt/wt | k*/k* | at/at | B/B | B/bs | B/B | D/D | S/S | h/h |
| **AE939** | **Australian Shepherd** | E/E | wt/wt | wt/wt | wt/wt | k*/k* | at/at | B/B | B/bs | B/B | D/D | S/S | h/h |
| **AE941** | **French Bulldog** | E/E | Em/wt | wt/wt | wt/wt | K/k* | Ay/a | B/B | B/B | B/B | D/d | S/S | h/h |
| **AE942** | **French Bulldog** | E/E | Em/Em | wt/wt | wt/wt | K/k* | Ay/a | B/B | B/B | B/B | d/d | S/sp | h/h |
| **AE943** | **Australian Shepherd** | E/e | Em/wt | wt/wt | wt/wt | k*/k* | at/at | B/B | bs/bs | B/B | D/D | S/S | h/h |
| **AE956** | **Catahoula** | E/E | Em/wt | wt/wt | wt/wt | K/k* | at/at | B/B | B/B | B/B | D/d | S/S | h/h |
| **AE957** | **Australian Shepherd** | E/E | Em/wt | wt/wt | wt/wt | k*/k* | at/at | B/B | B/bs | B/B | D/D | S/S | h/h |
| **AE981** | **Shetland Sheepdog** | E/E | wt/wt | wt/wt | wt/wt | k*/k* | Ay/aw | B/B | B/B | B/B | D/D | S/S | h/h |
| **AE982** | **Shetland Sheepdog** | E/E | wt/wt | wt/wt | wt/wt | k*/k* | at/a | B/B | B/B | B/B | D/D | S/S | h/h |
| **AF008** | **Australian Shepherd** | E/E | wt/wt | wt/wt | wt/wt | k*/k* | at/at | B/bc | B/B | B/bd | D/D | S/S | h/h |
| **AF009** | **Australian Shepherd** | E/E | wt/wt | wt/wt | wt/wt | k*/k* | at/at | B/bc | B/B | B/bd | D/d | S/S | h/h |
| **AF010** | **Australian Shepherd** | E/E | wt/wt | wt/wt | wt/wt | k*/k* | at/at | B/bc | B/B | B/bd | D/d | S/S | h/h |
| **AF011** | **Australian Shepherd** | E/E | Em/wt | wt/wt | wt/wt | k*/k* | at/at | B/bc | B/B | B/bd | D/D | S/S | h/h |
| **AF012** | **Australian Shepherd** | E/E | wt/wt | wt/wt | wt/wt | k*/k* | at/at | B/bc | B/B | B/bd | D/D | S/S | h/h |
| **AF013** | **Australian Shepherd** | E/E | wt/wt | wt/wt | wt/wt | k*/k* | at/at | bc/bc | B/B | B/bd | D/d | S/S | h/h |
| **AF014** | **Australian Shepherd** | E/E | Em/wt | wt/wt | wt/wt | k*/k* | at/at | B/B | B/B | B/B | D/D | S/S | h/h |
| **AF015** | **Australian Shepherd** | E/E | Em/wt | wt/wt | wt/wt | k*/k* | at/at | B/bc | B/B | B/bd | D/D | S/S | h/h |
| **AF016** | **Dachshund** | E/E | wt/wt | wt/wt | wt/wt | k*/k* | at/at | B/B | B/B | B/B | D/D | S/S | h/h |
| **AF017** | **Dachshund** | E/E | wt/wt | wt/wt | wt/wt | k*/k* | at/at | bc/bc | B/B | B/B | D/d | S/S | h/h |
| **AF018** | **Dachshund** | E/E | wt/wt | wt/wt | wt/wt | k*/k* | at/at | B/B | B/B | B/B | D/D | S/S | h/h |
| **AF019** | **Dachshund** | E/E | wt/wt | wt/wt | wt/wt | k*/k* | at/at | B/bc | B/B | B/B | D/D | S/S | h/h |
| **AF020** | **Dachshund** | E/E | wt/wt | wt/wt | wt/wt | k*/k* | at/at | B/bc | B/B | B/B | D/D | S/S | h/h |
| **AF022** | **Dachshund** | E/E | wt/wt | wt/wt | wt/wt | k*/k* | at/at | B/bc | B/B | B/B | D/d | S/S | h/h |
| **AF023** | **Dachshund** | E/E | wt/wt | wt/wt | wt/wt | k*/k* | at/at | B/bc | B/B | B/B | D/D | S/S | h/h |
| **AF038** | **Catahoula** | E/E | Em/Em | wt/wt | wt/wt | K/k* | aw/at | B/B | B/B | B/B | D/D | S/S | h/h |
| **AF039** | **Catahoula** | E/E | Em/Em | wt/wt | wt/wt | K/k* | Ay/a | B/B | B/B | B/B | D/D | S/S | h/h |
| **AF050** | **Australian Shepherd** | E/E | Em/wt | wt/wt | wt/wt | k*/k* | at/at | B/bc | B/B | B/bd | D/D | S/sp | h/h |
| **AF052** | **Australian Shepherd** | E/E | wt/wt | wt/wt | wt/wt | k*/k* | at/at | B/bc | B/B | B/bd | D/D | S/sp | h/h |
| **AF053** | **Catahoula** | E/E | wt/wt | wt/wt | wt/wt | K/k* | aw/at | B/B | B/bs | B/B | D/D | S/S | h/h |
| **AF056** | **Border Collie** | E/E | wt/wt | wt/wt | wt/wt | k*/k* | at/at | B/B | B/B | B/B | D/d | S/S | h/h |
| **AF072** | **Catahoula** | E/E | Em/wt | wt/wt | wt/wt | k*/k* | at/at | B/B | B/bs | B/B | D/d | S/S | h/h |
| **AF073** | **Rough Collie** | E/E | wt/wt | wt/wt | wt/wt | k*/k* | at/at | B/B | B/B | B/B | D/D | S/S | h/h |
| **AF074** | **Australian Shepherd** | E/E | wt/wt | wt/wt | wt/wt | K/k* | at/at | B/B | B/bs | B/B | D/D | S/S | h/h |
| **AF096** | **Australian Shepherd** | E/E | wt/wt | wt/wt | wt/wt | k*/k* | at/at | B/B | B/bs | B/B | D/D | S/sp | h/h |
| **AF099** | **Catahoula** | E/E | Em/Em | wt/wt | wt/wt | k*/k* | Ay/at | B/B | B/bs | B/B | D/D | S/S | h/h |
| **AF101** | **Catahoula** | E/E | Em/Em | wt/wt | wt/wt | K/k* | at/at | B/B | B/Bs | B/B | D/D | S/S | h/h |
| **AF104** | **French Bulldog** | E/E | Em/wt | wt/wt | wt/wt | K/k* | Ay/a | B/B | B/B | B/B | D/d | S/S | h/h |
| **AF129** | **Catahoula** | E/E | Em/Em | wt/wt | wt/wt | K/k* | at/at | B/B | B/bs | B/B | D/D | S/sp | h/h |
| **AF133** | **Catahoula** | E/E | Em/Em | wt/wt | wt/wt | K/k* | at/at | B/B | B/bs | B/B | D/D | S/S | h/h |
| **AF134** | **Border Collie** | E/e | Em/wt | wt/wt | wt/wt | K/K | aw/at | B/B | B/Bs | B/B | D/D | S/S | h/h |
| **AF135** | **Dachshund** | E/E | wt/wt | wt/wt | wt/wt | k*/k* | Ay/at | B/B | B/B | B/B | D/D | S/sp | h/h |
| **AF143** | **Australian Koolie** | E/E | Em/Em | wt/wt | wt/wt | K/k* | aw/at | B/B | bs/bs | B/B | D/D | S/S | h/h |
| **AF146** | **Catahoula** | E/E | Em/wt | wt/wt | wt/wt | K/k* | at/at | B/B | B/bs | B/B | D/D | S/sp | h/h |
| **AF161** | **Catahoula** | E/E | Em/Em | wt/wt | wt/wt | K/k* | at/at | B/B | bs/bs | B/B | D/d | S/sp | h/h |
| **AF163** | **Border Collie** | E/E | Em/wt | wt/wt | wt/wt | K/k* | at/at | B/B | B/B | B/B | D/D | S/S | h/h |
| **AF174** | **Koolie** | E/E | Em/wt | wt/wt | wt/wt | K/k* | at/at | B/B | B/B | B/B | D/D | S/S | h/h |
| **AF176** | **Catahoula** | E/E | Em/Em | wt/wt | wt/wt | K/k* | at/at | B/B | B/B | B/B | D/D | S/S | h/h |
| **AF185** | **Catahoula** | E/E | Em/Em | wt/wt | wt/wt | k*/k* | at/at | B/B | B/B | B/B | D/D | S/S | h/h |
| **AF195** | **Rough Collie** | E/E | wt/wt | wt/wt | wt/wt | k*/k* | at/at | B/B | B/B | B/B | D/D | S/sp | h/h |
| **AF196** | **Catahoula** | E/E | Em/wt | wt/wt | wt/wt | k*/k* | at/at | B/B | B/bs | B/B | D/D | S/S | h/h |
| **AF198** | **Catahoula** | E/E | Em/wt | wt/wt | wt/wt | k*/k* | at/at | B/B | bs/bs | B/B | D/D | S/S | h/h |
| **AF223** | **Australian Koolie** | E/E | wt/wt | wt/wt | wt/wt | k*/k* | at/at | B/B | bs/bs | B/B | D/d | S/S | h/h |
| **AF225** | **Australian Koolie** | E/E | wt/wt | wt/wt | wt/wt | K/K | at/at | B/B | B/bs | B/B | D/D | S/S | h/h |
| **AF227** | **Australian Koolie** | E/E | Em/wt | wt/wt | wt/wt | k*/k* | at/at | B/B | B/bs | B/B | D/D | S/S | h/h |
| **AF231** | **Catahoula** | E/e | Em/wt | wt/wt | wt/wt | k*/k* | at/a | B/B | B/bs | B/B | D/D | S/S | h/h |
| **AF232** | **Catahoula** | E/E | Em/Em | wt/wt | wt/wt | k*/k* | at/at | B/B | B/B | B/B | D/D | S/S | h/h |
| **AF237** | **Australian Shepherd** | E/E | Em/wt | wt/wt | wt/wt | k*/k* | at/at | B/bc | B/B | B/bd | D/D | S/S | h/h |
| **AF239** | **Australian Shepherd** | E/E | Em/wt | wt/wt | wt/wt | k*/k* | at/at | B/B | B/bs | B/B | D/D | S/S | h/h |
| **AF241** | **Australian Shepherd** | E/E | Em/Em | wt/wt | wt/wt | k*/k* | at/at | B/B | B/B | B/B | D/D | S/S | h/h |
| **AF265** | **Catahoula** | E/E | Em/Em | wt/wt | wt/wt | k*/k* | at/at | B/B | B/bs | B/B | D/D | S/S | h/h |
| **AF267** | **Miniature Australian Shepherd** | E/E | wt/wt | wt/wt | wt/wt | k*/k* | at/at | B/bc | B/B | B/bd | D/D | S/S | h/h |
| **AF270** | **Labradoodle** | E/e | Em/wt | wt/wt | wt/wt | K/k* | Ay/at | B/bc | B/B | B/bd | D/D | S/S | h/h |
| **AF273** | **Catahoula** | E/E | Em/wt | wt/wt | wt/wt | K/k* | at/at | B/B | bs/bs | B/B | D/D | S/sp | h/h |
| **AF274** | **Catahoula** | E/E | wt/wt | wt/wt | wt/wt | k*/k* | at/at | B/B | B/bs | B/B | D/D | S/S | h/h |
| **AF327** | **Australian Koolie** | E/E | Em/wt | wt/wt | wt/wt | k*/k* | at/at | B/B | B/bs | B/B | D/D | S/S | h/h |
| **AF330** | **Catahoula** | E/E | Em/wt | wt/wt | wt/wt | K/k* | at/at | B/B | B/bs | B/B | D/D | S/S | h/h |
| **AF331** | **Catahoula** | E/E | Em/wt | wt/wt | wt/wt | K/k* | at/at | B/B | bs/bs | B/B | D/D | S/S | h/h |
| **AF332** | **Catahoula** | E/E | wt/wt | wt/wt | wt/wt | K/k* | at/at | B/B | bs/bs | B/B | D/D | S/S | h/h |
| **AF333** | **Catahoula** | E/E | wt/wt | wt/wt | wt/wt | K/k* | at/at | B/B | B/bs | B/B | D/D | S/S | h/h |
| **AF334** | **Catahoula** | E/E | wt/wt | wt/wt | wt/wt | k*/k* | at/at | B/B | bs/bs | B/B | D/D | S/S | h/h |
| **AF335** | **Catahoula** | E/E | wt/wt | wt/wt | wt/wt | K/k* | at/at | B/B | bs/bs | B/B | D/D | S/S | h/h |
| **AF341** | **Catahoula** | E/E | wt/wt | wt/wt | wt/wt | K/k* | at/at | B/B | B/bs | B/B | D/d | S/S | h/h |
| **AF350** | **Australian Koolie** | E/E | wt/wt | wt/wt | wt/wt | k*/k* | aw/at | B/B | B/bs | B/B | D/d | S/sp | h/h |
| **AF351** | **Australian Koolie** | E/E | Em/wt | wt/wt | wt/wt | K/k* | at/at | B/B | bs/bs | B/B | D/d | S/sp | h/h |
| **AF352** | **Catahoula** | E/E | wt/wt | wt/wt | wt/wt | K/k* | aw/at | B/B | B/B | B/B | D/D | S/S | h/h |
| **AF378** | **Catahoula** | E/E | Em/wt | wt/wt | wt/wt | k*/k* | at/at | B/B | B/bs | B/B | D/D | S/S | h/h |
| **AF379** | **Catahoula** | E/E | Em/wt | wt/wt | wt/wt | k*/k* | at/at | B/B | B/bs | B/B | D/D | S/S | h/h |
| **AF380** | **Catahoula** | E/E | wt/wt | wt/wt | wt/wt | k*/k* | at/at | B/B | bs/bs | B/B | D/D | S/S | h/h |
| **AF381** | **Border Collie** | E/E | Em/wt | wt/wt | wt/wt | k*/k* | at/at | B/B | B/bs | B/B | D/D | S/S | h/h |
| **AF382** | **Border Collie** | E/E | Em/Em | wt/wt | wt/wt | K/k* | aw/at | B/B | B/B | B/B | D/d | S/S | h/h |
| **AF398** | **Australian Shepherd** | E/E | Em/Em | wt/wt | wt/wt | k*/k* | aw/at | B/bc | B/B | B/bd | D/D | S/S | h/h |
| **AF399** | **Catahoula** | E/E | Em/wt | wt/wt | wt/wt | K/k* | at/at | B/B | bs/bs | B/B | D/D | S/sp | h/h |
| **AF400** | **Australian Koolie** | E/E | Em/wt | wt/wt | wt/wt | K/k* | aw/at | B/B | bs/bs | B/B | D/d | S/S | h/h |
| **AF401** | **Rough Collie** | E/E | wt/wt | wt/wt | wt/wt | k*/k* | at/at | B/B | B/B | B/B | D/D | S/sp | h/h |
| **AF402** | **Catahoula** | E/e | wt/wt | wt/wt | wt/wt | k*/k* | at/a | B/B | B/bs | B/B | D/D | S/S | h/h |
| **AF425** | **Catahoula** | E/E | Em/wt | wt/wt | wt/wt | k*/k* | at/at | B/B | B/B | B/B | D/D | S/S | h/h |
| **AF426** | **Catahoula** | E/E | Em/Em | wt/wt | wt/wt | k*/k* | at/at | B/B | B/bs | B/B | D/d | S/S | h/h |
| **AF431** | **Border Collie** | E/E | wt/wt | wt/wt | wt/wt | K/k* | at/at | B/B | bs/bs | B/B | D/D | S/S | h/h |
| **AF432** | **Australian Koolie** | E/E | Em/wt | wt/wt | wt/wt | K/k* | Ay/at | B/bc | B/bs | B/bd | D/D | S/S | h/h |
| **AF457** | **Australian Shepherd** | E/E | Em/wt | wt/wt | wt/wt | k*/k* | at/at | B/B | bs/bs | B/B | D/D | S/S | h/h |
| **AF459** | **Border Collie** | E/E | wt/wt | wt/wt | wt/wt | K/K | at/at | B/B | bs/bs | B/B | D/d | S/S | h/h |
| **AF460** | **Australian Koolie** | E/E | Em/wt | wt/wt | wt/wt | K/K | at/at | B/B | B/bs | B/B | D/D | S/S | h/h |
| **AF462** | **Catahoula** | E/E | Em/wt | wt/wt | wt/wt | K/k* | at/at | B/B | B/bs | B/B | D/D | S/S | h/h |
| **AF472** | **Australian Shepherd** | E/E | Em/wt | wt/wt | wt/wt | k*/k* | at/at | B/bc | B/B | B/bd | D/D | S/sp | h/h |
| **AF473** | **Border Collie** | E/E | Em/Em | wt/wt | wt/wt | K/k* | aw/at | B/B | B/bs | B/B | D/D | S/S | h/h |
| **AF474** | **Australian Shepherd** | E/E | wt/wt | wt/wt | wt/wt | k*/k* | at/a | B/bc | B/bs | B/bd | D/D | S/S | h/h |
| **AF475** | **Australian Shepherd** | E/E | Em/wt | wt/wt | wt/wt | K/k* | at/at | B/bc | B/B | B/bd | D/D | S/S | h/h |
| **AF480** | **Australian Koolie** | E/E | wt/wt | wt/wt | wt/wt | K/k* | at/at | B/bc | B/bs | B/bd | d/d | S/S | h/h |
| **AF481** | **Australian Koolie** | E/E | wt/wt | wt/wt | wt/wt | K/k* | at/at | B/B | bs/bs | B/B | D/d | S/S | h/h |
| **AF482** | **Australian Koolie** | E/E | Em/wt | wt/wt | wt/wt | K/k* | aw/at | B/B | B/bs | B/B | d/d | S/S | h/h |
| **AF483** | **Australian Koolie** | E/E | Em/wt | wt/wt | wt/wt | k*/k* | at/at | B/B | B/bs | B/B | D/d | S/S | h/h |
| **AF484** | **Australian Koolie** | E/E | Em/wt | wt/wt | wt/wt | k*/k* | at/at | B/B | bs/bs | B/B | D/d | S/S | h/h |
| **AF485** | **Australian Koolie** | E/E | Em/wt | wt/wt | wt/wt | k*/k* | at/at | B/B | B/bs | B/B | D/D | S/S | h/h |
| **AF509** | **Australian Shepherd** | E/E | Em/wt | wt/wt | wt/wt | k*/k* | at/at | B/B | B/bs | B/B | D/D | S/sp | h/h |
| **AF510** | **Catahoula** | E/E | Em/wt | wt/wt | wt/wt | k*/k* | at/a | B/B | bs/bs | B/B | D/D | S/S | h/h |
| **AF511** | **Catahoula** | E/E | Em/wt | wt/wt | wt/wt | k*/k* | at/a | B/B | B/bs | B/B | D/D | S/S | h/h |
| **AF512** | **Catahoula** | E/E | Em/Em | wt/wt | wt/wt | k*/k* | at/a | B/B | B/B | B/B | D/D | S/S | h/h |
| **AF513** | **Australian Koolie** | E/E | Em/Em | wt/wt | wt/wt | K/k* | at/at | B/B | B/bs | B/B | D/d | S/S | h/h |
| **AF514** | **Border Collie** | E/E | Em/wt | wt/wt | wt/wt | K/k* | at/at | B/B | B/B | B/B | D/D | S/S | h/h |
| **AF515** | **Catahoula** | E/E | wt/wt | wt/wt | wt/wt | K/k* | at/at | B/B | B/bs | B/B | D/D | S/S | h/h |
| **AF521** | **Australian Koolie** | E/E | Em/Em | wt/wt | wt/wt | K/k* | at/at | B/B | B/bs | B/B | D/d | S/S | h/h |
| **AF522** | **Border Collie** | E/E | wt/wt | wt/wt | wt/wt | K/K | aw/at | B/bc | B/bs | B/bd | D/[d]/d | S/sp | h/h |
| **AF523** | **Pyrenean Shepherd** | E/E | Em/Em | wt/wt | wt/wt | K/k* | aw/aw | B/B | B/B | B/B | D/D | S/S | h/h |
| **AF524** | **Australian Shepherd** | E/e | wt/wt | wt/wt | wt/wt | k*/k* | at/at | B/B | B/B | B/B | D/D | S/S | h/h |
| **AF525** | **Catahoula** | E/E | Em/wt | wt/wt | wt/wt | k*/k* | at/at | B/B | B/bs | B/B | D/d | S/S | h/h |
| **AF578** | **Dachshund** | E/E | wt/wt | wt/wt | wt/wt | k*/k* | at/at | B/B | bs/bs | B/B | D/D | S/S | h/h |
| **AF579** | **Border Collie** | E/E | wt/wt | wt/wt | wt/wt | K/k* | at/at | B/B | B/bs | B/B | D/D | S/S | h/h |
| **AF580** | **Australian Koolie** | E/E | Em/wt | wt/wt | wt/wt | K/k* | at/at | B/B | B/bs | B/B | D/D | S/sp | h/h |
| **AF612** | **Catahoula** | E/E | wt/wt | wt/wt | wt/wt | K/k* | at/at | B/B | bs/bs | B/B | D/D | S/sp | h/h |
| **AF613** | **Welsh Sheepdog** | E/E | Em/wt | wt/wt | wt/wt | k*/k* | Ay/at | B/B | B/B | B/B | D/D | S/sp | h/h |
| **AF614** | **Welsh Sheepdog** | E/E | Em/wt | wt/wt | wt/wt | k*/k* | Ay/at | B/B | B/B | B/B | D/D | S/S | h/h |
| **AF615** | **Welsh Sheepdog** | E/E | Em/wt | wt/wt | wt/wt | K/k* | Ay/Ay | B/B | B/B | B/B | D/D | S/sp | h/h |
| **AF649** | **Mudi** | E/e | wt/wt | wt/wt | wt/wt | K/k* | a/a | B/B | B/B | B/B | D/D | S/S | h/h |
| **AF650** | **Mudi** | e/e | wt/wt | wt/wt | wt/wt | K/k* | a/a | B/B | B/B | B/B | D/D | S/S | h/h |
| **AF652** | **Australian Shepherd** | E/E | wt/wt | wt/wt | wt/wt | k*/k* | aw/at | B/bc | B/B | B/bd | D/D | S/S | h/h |
| **AF658** | **Australian Shepherd** | E/E | wt/wt | wt/wt | wt/wt | k*/k* | at/at | B/bc | B/B | B/bd | D/D | S/S | h/h |
| **AF668** | **Australian Shepherd** | E/E | wt/wt | wt/wt | wt/wt | k*/k* | at/at | B/bc | B/B | B/bd | D/D | S/S | h/h |
| **AF669** | **Catahoula** | E/E | Em/Em | wt/wt | wt/wt | K/k* | at/a | B/B | B/B | B/B | D/D | S/S | h/h |
| **AF694** | **Catahoula** | E/E | Em/wt | wt/wt | wt/wt | K/k* | at/at | B/B | B/B | B/B | D/D | S/sp | h/h |
| **AF696** | **Australian Koolie** | E/E | Em/Em | wt/wt | wt/wt | K/k* | at/at | B/B | B/bs | B/B | D/D | S/S | h/h |
| **AF697** | **Australian Koolie** | E/E | Em/wt | wt/wt | wt/wt | K/K | at/at | B/B | B/bs | B/B | D/D | S/S | h/h |
| **AF699** | **Catahoula** | E/E | wt/wt | wt/wt | wt/wt | K/k* | at/at | B/B | B/B | B/B | D/D | S/S | h/h |
| **AF758** | **Australina Shepherd** | E/E | wt/wt | wt/wt | wt/wt | K/k* | at/at | B/bc | B/B | B/bd | D/d | S/S | h/h |
| **AF759** | **Australian Shepherd** | E/E | wt/wt | wt/wt | wt/wt | K/k* | at/at | B/B | B/bs | B/B | D/d | S/sp | h/h |
| **AF760** | **Australian Shepherd** | E/E | wt/wt | wt/wt | wt/wt | k*/k* | at/at | B/bc | B/B | B/bd | D/D | S/sp | h/h |
| **AF761** | **Australian Shepherd** | E/E | wt/wt | wt/wt | wt/wt | K/k* | at/at | B/B | B/B | B/B | D/d | S/S | h/h |
| **AF762** | **Australian Shepherd** | E/E | wt/wt | wt/wt | wt/wt | k*/k* | at/at | B/bc | B/bs | B/bd | D/D | S/sp | h/h |
| **AF778** | **Border Collie** | E/E | Em/wt | wt/wt | wt/wt | K/k* | at/a | B/B | B/bs | B/B | D/D | S/sp | h/h |
| **AF781** | **Border Collie** | E/e | wt/wt | wt/wt | wt/wt | K/k* | at/at | B/B | B/bs | B/B | D/D | S/S | h/h |
| **AF782** | **Border Collie** | E/E | wt/wt | wt/wt | wt/wt | K/K | at/at | B/B | B/bs | B/B | D/D | S/S | h/h |
| **AF824** | **Australian Koolie** | E/E | wt/wt | wt/wt | wt/wt | K/k* | at/at | B/B | bs/bs | B/B | D/D | S/S | h/h |
