# Supplementary material for: Merle phenotypes in dogs – SILV SINE insertions from Mc to Mh
Source: PLoS One. 2018 Sep 20;13(9):e0198536. doi: 10.1371/journal.pone.0198536 (PMC6147463; doi:10.1371/journal.pone.0198536)
Supplement: S1 File — The Mh allele has a broad range of phenotypes with 2 expressions that are very recognizable. (DOCX) [file pone.0198536.s006.docx]

**Genotype/phenotype correlations: Mh.** The Mh allele has a broad range of phenotypes with 2 expressions that are very recognizable. A - “Minimal Merle” - a large percentage of the body features solid colored pigment with only small random areas of Merle patterning. Individuals may also express extended white out of the normal area of the typical Irish Spotting pattern – this may include a large white collar, white up legs past the elbow, white past shoulders extending onto withers and white on the belly extending up the side. This extended white is sometimes associated with S/sp - (Piebald Carrier), however many m/Mh dogs with this type of white pattern have tested as S/S.

B - The more classic pattern that is often referred to as “Herding Harlequin” - Random diluted areas of Merle pigment are deleted to white, leaving solid patched areas that may be Tweed patterned including different shades. Some Merle areas may remain. The extended white patterning mentioned in description #1 may be present but is less noticeable due to the deleted white areas on the body.

C - Some dogs may express more as m/M, yet are still able to produce offspring with a phenotype as described above in example A and B - these offspring have inherited the same length of base pairs as the parent and yet express in either of the 3 ways presented here. For more details see S2 Fig.
