# Supplementary figures and images for: Merle phenotypes in dogs – SILV SINE insertions from Mc to Mh
Source: PLoS One. 2018 Sep 20;13(9):e0198536. doi: 10.1371/journal.pone.0198536 (PMC6147463; doi:10.1371/journal.pone.0198536)

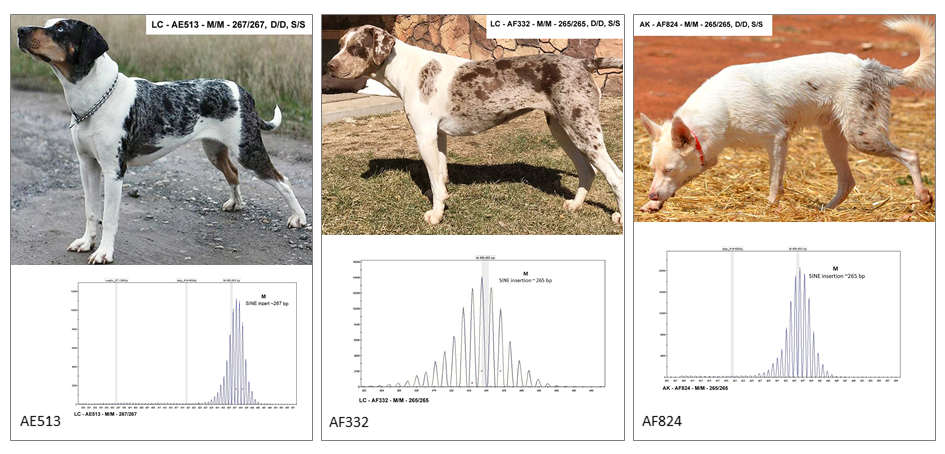

Supplement: S1 Fig — , subdivided into Figures A-X in S1 Fig according to the individual Merle allelic combinations, shows photographs and respective Merle allelic chromatograms of all 181 dogs included in our study. For detailed description of the major phenotypic features associated with the individual genotypes please refer to the main body of the article that can be found in Fig 3. The individual subdivisions of S1 Fig are the following: Figure A in S1 Fig Genotype/phenotype correlations: m/m; Figure B in S1 Fig Genotype/phenotype correlations: m/Mc; Figure C in S1 Fig Genotype/phenotype correlations: Mc/Mc; Figure D in S1 Fig Genotype / phenotype correlations: m/Mc+; Figure E in S1 Fig Genotype/phenotype correlations: Mc+/Mc+; Figure F in S1 Fig Genotype/phenotype correlations: m/Ma; Figure G in S1 Fig Genotype/phenotype correlations: Mc/Ma; Figure H in S1 Fig Genotype/phenotype correlations: Ma/Ma; Figure I in S1 Fig Genotype/phenotype correlations: m/Ma+; Figure J in S1 Fig Genotype/phenotype correlations: Mc/Ma+; Figure K in S1 Fig Genotype/phenotype correlations: Mc+/Ma+; Figure L in S1 Fig Genotype/phenotype correlations: Ma+/Ma+; Figure M in S1 Fig Genotype/phenotype correlations: m/M; Figure N in S1 Fig Genotype/phenotype correlations: Mc/M; Figure O in S1 Fig Genotype/phenotype correlations: Mc+/M; Figure P in S1 Fig Genotype/phenotype correlations: Ma/M; Figure Q in S1 Fig Genotype/phenotype correlations: Ma+/M; Figure R in S1 Fig Genotype/phenotype correlations: M/M; Figure S in S1 Fig Genotype/phenotype correlations: m/Mh; Figure T in S1 Fig Genotype/phenotype correlations: Mc/Mh; Figure U in S1 Fig Genotype/phenotype correlations: Mc+/Mh; Figure V in S1 Fig Genotype/phenotype correlations: Ma/Mh; Figure W in S1 Fig Genotype/phenotype correlations: M/Mh; Figure X in S1 Fig Genotype/phenotype correlations: Mh/Mh. (ZIP) [file pone.0198536.s007.zip › Figure R in S1 Fig_Langevin et al.tif]

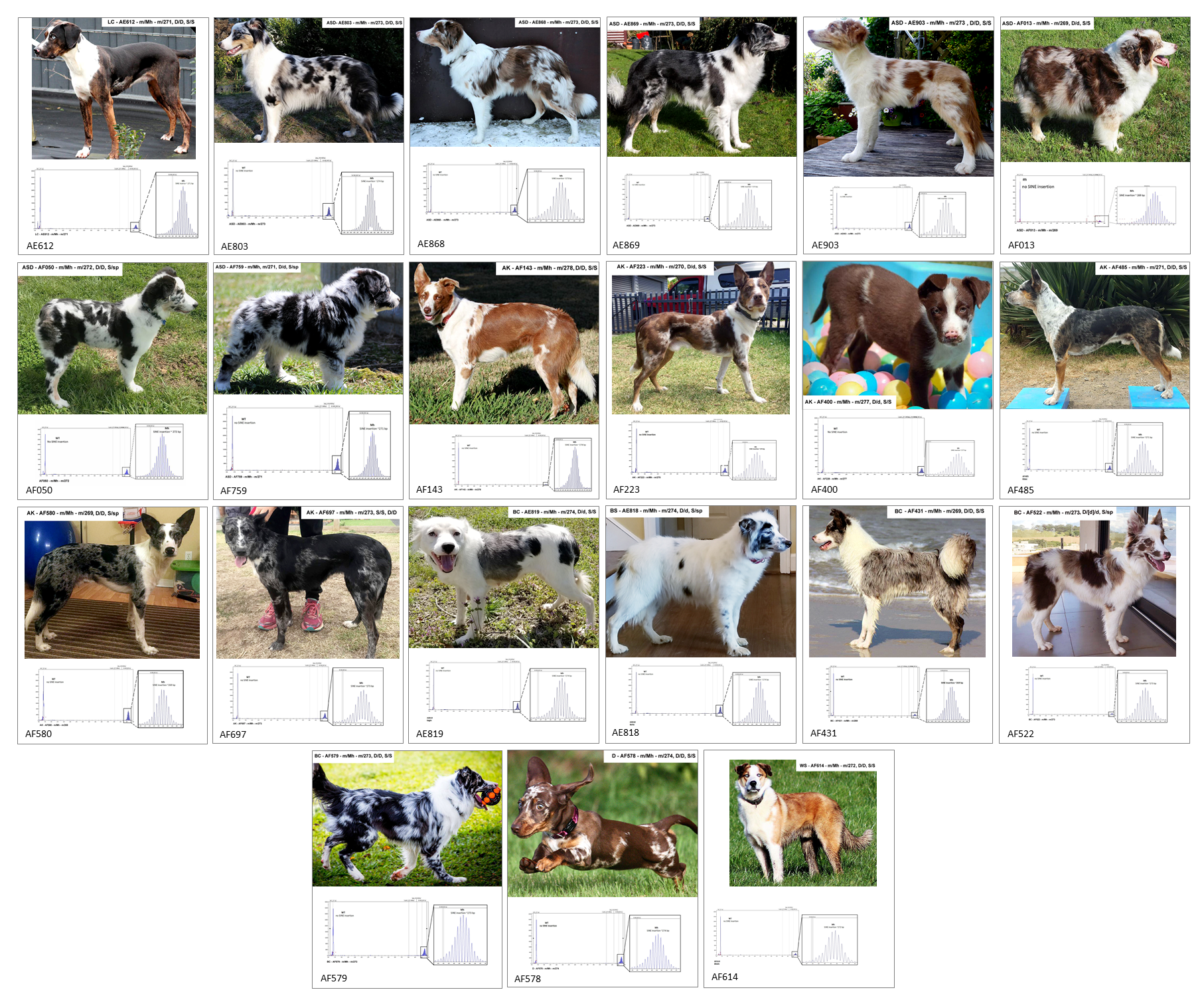

Supplement: S1 Fig — , subdivided into Figures A-X in S1 Fig according to the individual Merle allelic combinations, shows photographs and respective Merle allelic chromatograms of all 181 dogs included in our study. For detailed description of the major phenotypic features associated with the individual genotypes please refer to the main body of the article that can be found in Fig 3. The individual subdivisions of S1 Fig are the following: Figure A in S1 Fig Genotype/phenotype correlations: m/m; Figure B in S1 Fig Genotype/phenotype correlations: m/Mc; Figure C in S1 Fig Genotype/phenotype correlations: Mc/Mc; Figure D in S1 Fig Genotype / phenotype correlations: m/Mc+; Figure E in S1 Fig Genotype/phenotype correlations: Mc+/Mc+; Figure F in S1 Fig Genotype/phenotype correlations: m/Ma; Figure G in S1 Fig Genotype/phenotype correlations: Mc/Ma; Figure H in S1 Fig Genotype/phenotype correlations: Ma/Ma; Figure I in S1 Fig Genotype/phenotype correlations: m/Ma+; Figure J in S1 Fig Genotype/phenotype correlations: Mc/Ma+; Figure K in S1 Fig Genotype/phenotype correlations: Mc+/Ma+; Figure L in S1 Fig Genotype/phenotype correlations: Ma+/Ma+; Figure M in S1 Fig Genotype/phenotype correlations: m/M; Figure N in S1 Fig Genotype/phenotype correlations: Mc/M; Figure O in S1 Fig Genotype/phenotype correlations: Mc+/M; Figure P in S1 Fig Genotype/phenotype correlations: Ma/M; Figure Q in S1 Fig Genotype/phenotype correlations: Ma+/M; Figure R in S1 Fig Genotype/phenotype correlations: M/M; Figure S in S1 Fig Genotype/phenotype correlations: m/Mh; Figure T in S1 Fig Genotype/phenotype correlations: Mc/Mh; Figure U in S1 Fig Genotype/phenotype correlations: Mc+/Mh; Figure V in S1 Fig Genotype/phenotype correlations: Ma/Mh; Figure W in S1 Fig Genotype/phenotype correlations: M/Mh; Figure X in S1 Fig Genotype/phenotype correlations: Mh/Mh. (ZIP) [file pone.0198536.s007.zip › Figure S in S1 Fig_Langevin et al.tif]

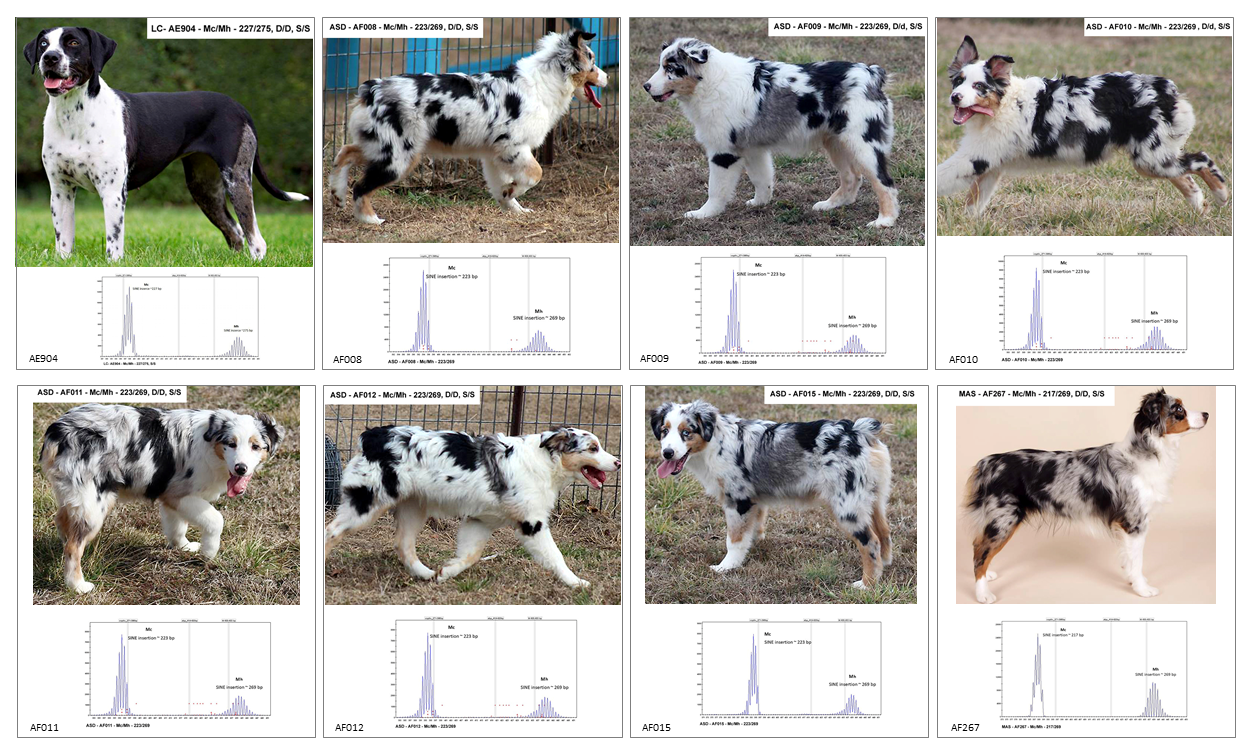

Supplement: S1 Fig — , subdivided into Figures A-X in S1 Fig according to the individual Merle allelic combinations, shows photographs and respective Merle allelic chromatograms of all 181 dogs included in our study. For detailed description of the major phenotypic features associated with the individual genotypes please refer to the main body of the article that can be found in Fig 3. The individual subdivisions of S1 Fig are the following: Figure A in S1 Fig Genotype/phenotype correlations: m/m; Figure B in S1 Fig Genotype/phenotype correlations: m/Mc; Figure C in S1 Fig Genotype/phenotype correlations: Mc/Mc; Figure D in S1 Fig Genotype / phenotype correlations: m/Mc+; Figure E in S1 Fig Genotype/phenotype correlations: Mc+/Mc+; Figure F in S1 Fig Genotype/phenotype correlations: m/Ma; Figure G in S1 Fig Genotype/phenotype correlations: Mc/Ma; Figure H in S1 Fig Genotype/phenotype correlations: Ma/Ma; Figure I in S1 Fig Genotype/phenotype correlations: m/Ma+; Figure J in S1 Fig Genotype/phenotype correlations: Mc/Ma+; Figure K in S1 Fig Genotype/phenotype correlations: Mc+/Ma+; Figure L in S1 Fig Genotype/phenotype correlations: Ma+/Ma+; Figure M in S1 Fig Genotype/phenotype correlations: m/M; Figure N in S1 Fig Genotype/phenotype correlations: Mc/M; Figure O in S1 Fig Genotype/phenotype correlations: Mc+/M; Figure P in S1 Fig Genotype/phenotype correlations: Ma/M; Figure Q in S1 Fig Genotype/phenotype correlations: Ma+/M; Figure R in S1 Fig Genotype/phenotype correlations: M/M; Figure S in S1 Fig Genotype/phenotype correlations: m/Mh; Figure T in S1 Fig Genotype/phenotype correlations: Mc/Mh; Figure U in S1 Fig Genotype/phenotype correlations: Mc+/Mh; Figure V in S1 Fig Genotype/phenotype correlations: Ma/Mh; Figure W in S1 Fig Genotype/phenotype correlations: M/Mh; Figure X in S1 Fig Genotype/phenotype correlations: Mh/Mh. (ZIP) [file pone.0198536.s007.zip › Figure T in S1 Fig_Langevin et al.tif]

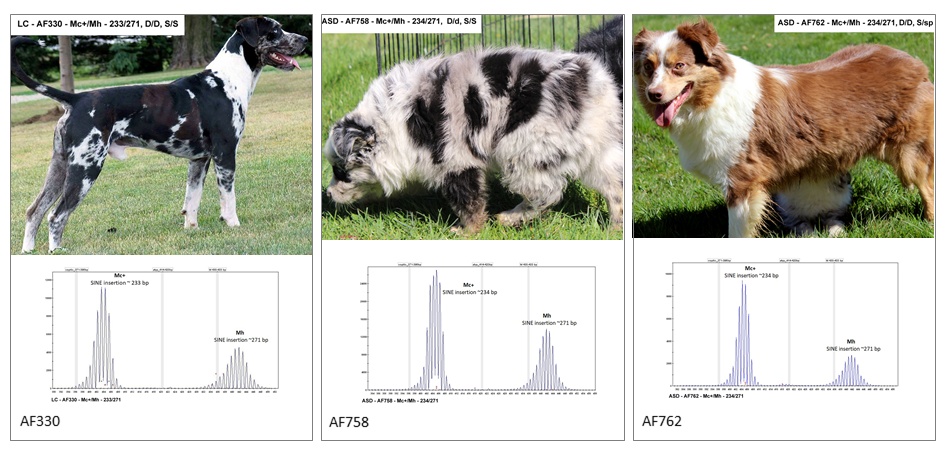

Supplement: S1 Fig — , subdivided into Figures A-X in S1 Fig according to the individual Merle allelic combinations, shows photographs and respective Merle allelic chromatograms of all 181 dogs included in our study. For detailed description of the major phenotypic features associated with the individual genotypes please refer to the main body of the article that can be found in Fig 3. The individual subdivisions of S1 Fig are the following: Figure A in S1 Fig Genotype/phenotype correlations: m/m; Figure B in S1 Fig Genotype/phenotype correlations: m/Mc; Figure C in S1 Fig Genotype/phenotype correlations: Mc/Mc; Figure D in S1 Fig Genotype / phenotype correlations: m/Mc+; Figure E in S1 Fig Genotype/phenotype correlations: Mc+/Mc+; Figure F in S1 Fig Genotype/phenotype correlations: m/Ma; Figure G in S1 Fig Genotype/phenotype correlations: Mc/Ma; Figure H in S1 Fig Genotype/phenotype correlations: Ma/Ma; Figure I in S1 Fig Genotype/phenotype correlations: m/Ma+; Figure J in S1 Fig Genotype/phenotype correlations: Mc/Ma+; Figure K in S1 Fig Genotype/phenotype correlations: Mc+/Ma+; Figure L in S1 Fig Genotype/phenotype correlations: Ma+/Ma+; Figure M in S1 Fig Genotype/phenotype correlations: m/M; Figure N in S1 Fig Genotype/phenotype correlations: Mc/M; Figure O in S1 Fig Genotype/phenotype correlations: Mc+/M; Figure P in S1 Fig Genotype/phenotype correlations: Ma/M; Figure Q in S1 Fig Genotype/phenotype correlations: Ma+/M; Figure R in S1 Fig Genotype/phenotype correlations: M/M; Figure S in S1 Fig Genotype/phenotype correlations: m/Mh; Figure T in S1 Fig Genotype/phenotype correlations: Mc/Mh; Figure U in S1 Fig Genotype/phenotype correlations: Mc+/Mh; Figure V in S1 Fig Genotype/phenotype correlations: Ma/Mh; Figure W in S1 Fig Genotype/phenotype correlations: M/Mh; Figure X in S1 Fig Genotype/phenotype correlations: Mh/Mh. (ZIP) [file pone.0198536.s007.zip › Figure U in S1 Fig_Langevin et al.tif]

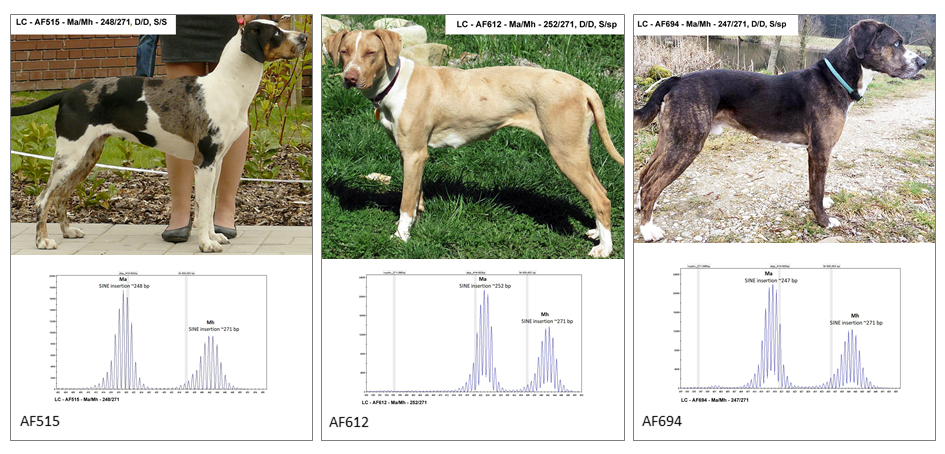

Supplement: S1 Fig — , subdivided into Figures A-X in S1 Fig according to the individual Merle allelic combinations, shows photographs and respective Merle allelic chromatograms of all 181 dogs included in our study. For detailed description of the major phenotypic features associated with the individual genotypes please refer to the main body of the article that can be found in Fig 3. The individual subdivisions of S1 Fig are the following: Figure A in S1 Fig Genotype/phenotype correlations: m/m; Figure B in S1 Fig Genotype/phenotype correlations: m/Mc; Figure C in S1 Fig Genotype/phenotype correlations: Mc/Mc; Figure D in S1 Fig Genotype / phenotype correlations: m/Mc+; Figure E in S1 Fig Genotype/phenotype correlations: Mc+/Mc+; Figure F in S1 Fig Genotype/phenotype correlations: m/Ma; Figure G in S1 Fig Genotype/phenotype correlations: Mc/Ma; Figure H in S1 Fig Genotype/phenotype correlations: Ma/Ma; Figure I in S1 Fig Genotype/phenotype correlations: m/Ma+; Figure J in S1 Fig Genotype/phenotype correlations: Mc/Ma+; Figure K in S1 Fig Genotype/phenotype correlations: Mc+/Ma+; Figure L in S1 Fig Genotype/phenotype correlations: Ma+/Ma+; Figure M in S1 Fig Genotype/phenotype correlations: m/M; Figure N in S1 Fig Genotype/phenotype correlations: Mc/M; Figure O in S1 Fig Genotype/phenotype correlations: Mc+/M; Figure P in S1 Fig Genotype/phenotype correlations: Ma/M; Figure Q in S1 Fig Genotype/phenotype correlations: Ma+/M; Figure R in S1 Fig Genotype/phenotype correlations: M/M; Figure S in S1 Fig Genotype/phenotype correlations: m/Mh; Figure T in S1 Fig Genotype/phenotype correlations: Mc/Mh; Figure U in S1 Fig Genotype/phenotype correlations: Mc+/Mh; Figure V in S1 Fig Genotype/phenotype correlations: Ma/Mh; Figure W in S1 Fig Genotype/phenotype correlations: M/Mh; Figure X in S1 Fig Genotype/phenotype correlations: Mh/Mh. (ZIP) [file pone.0198536.s007.zip › Figure V in S1 Fig_Langevin et al.tif]

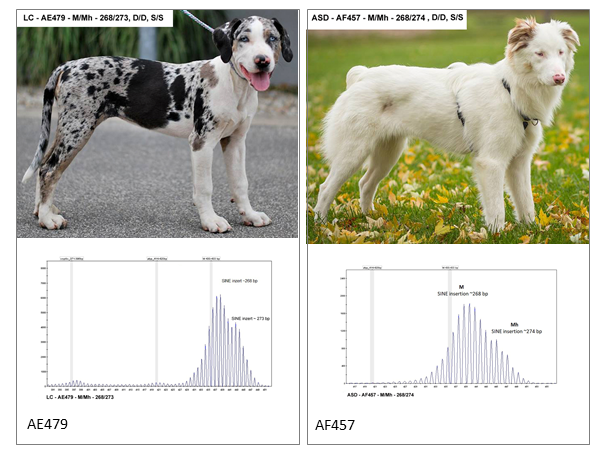

Supplement: S1 Fig — , subdivided into Figures A-X in S1 Fig according to the individual Merle allelic combinations, shows photographs and respective Merle allelic chromatograms of all 181 dogs included in our study. For detailed description of the major phenotypic features associated with the individual genotypes please refer to the main body of the article that can be found in Fig 3. The individual subdivisions of S1 Fig are the following: Figure A in S1 Fig Genotype/phenotype correlations: m/m; Figure B in S1 Fig Genotype/phenotype correlations: m/Mc; Figure C in S1 Fig Genotype/phenotype correlations: Mc/Mc; Figure D in S1 Fig Genotype / phenotype correlations: m/Mc+; Figure E in S1 Fig Genotype/phenotype correlations: Mc+/Mc+; Figure F in S1 Fig Genotype/phenotype correlations: m/Ma; Figure G in S1 Fig Genotype/phenotype correlations: Mc/Ma; Figure H in S1 Fig Genotype/phenotype correlations: Ma/Ma; Figure I in S1 Fig Genotype/phenotype correlations: m/Ma+; Figure J in S1 Fig Genotype/phenotype correlations: Mc/Ma+; Figure K in S1 Fig Genotype/phenotype correlations: Mc+/Ma+; Figure L in S1 Fig Genotype/phenotype correlations: Ma+/Ma+; Figure M in S1 Fig Genotype/phenotype correlations: m/M; Figure N in S1 Fig Genotype/phenotype correlations: Mc/M; Figure O in S1 Fig Genotype/phenotype correlations: Mc+/M; Figure P in S1 Fig Genotype/phenotype correlations: Ma/M; Figure Q in S1 Fig Genotype/phenotype correlations: Ma+/M; Figure R in S1 Fig Genotype/phenotype correlations: M/M; Figure S in S1 Fig Genotype/phenotype correlations: m/Mh; Figure T in S1 Fig Genotype/phenotype correlations: Mc/Mh; Figure U in S1 Fig Genotype/phenotype correlations: Mc+/Mh; Figure V in S1 Fig Genotype/phenotype correlations: Ma/Mh; Figure W in S1 Fig Genotype/phenotype correlations: M/Mh; Figure X in S1 Fig Genotype/phenotype correlations: Mh/Mh. (ZIP) [file pone.0198536.s007.zip › Figure W in S1 Fig_Langevin et al.tif]

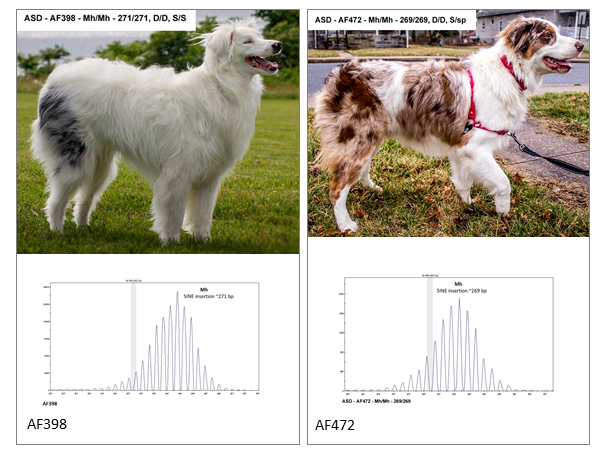

Supplement: S1 Fig — , subdivided into Figures A-X in S1 Fig according to the individual Merle allelic combinations, shows photographs and respective Merle allelic chromatograms of all 181 dogs included in our study. For detailed description of the major phenotypic features associated with the individual genotypes please refer to the main body of the article that can be found in Fig 3. The individual subdivisions of S1 Fig are the following: Figure A in S1 Fig Genotype/phenotype correlations: m/m; Figure B in S1 Fig Genotype/phenotype correlations: m/Mc; Figure C in S1 Fig Genotype/phenotype correlations: Mc/Mc; Figure D in S1 Fig Genotype / phenotype correlations: m/Mc+; Figure E in S1 Fig Genotype/phenotype correlations: Mc+/Mc+; Figure F in S1 Fig Genotype/phenotype correlations: m/Ma; Figure G in S1 Fig Genotype/phenotype correlations: Mc/Ma; Figure H in S1 Fig Genotype/phenotype correlations: Ma/Ma; Figure I in S1 Fig Genotype/phenotype correlations: m/Ma+; Figure J in S1 Fig Genotype/phenotype correlations: Mc/Ma+; Figure K in S1 Fig Genotype/phenotype correlations: Mc+/Ma+; Figure L in S1 Fig Genotype/phenotype correlations: Ma+/Ma+; Figure M in S1 Fig Genotype/phenotype correlations: m/M; Figure N in S1 Fig Genotype/phenotype correlations: Mc/M; Figure O in S1 Fig Genotype/phenotype correlations: Mc+/M; Figure P in S1 Fig Genotype/phenotype correlations: Ma/M; Figure Q in S1 Fig Genotype/phenotype correlations: Ma+/M; Figure R in S1 Fig Genotype/phenotype correlations: M/M; Figure S in S1 Fig Genotype/phenotype correlations: m/Mh; Figure T in S1 Fig Genotype/phenotype correlations: Mc/Mh; Figure U in S1 Fig Genotype/phenotype correlations: Mc+/Mh; Figure V in S1 Fig Genotype/phenotype correlations: Ma/Mh; Figure W in S1 Fig Genotype/phenotype correlations: M/Mh; Figure X in S1 Fig Genotype/phenotype correlations: Mh/Mh. (ZIP) [file pone.0198536.s007.zip › Figure X in S1 Fig_Langevin et al.tif]

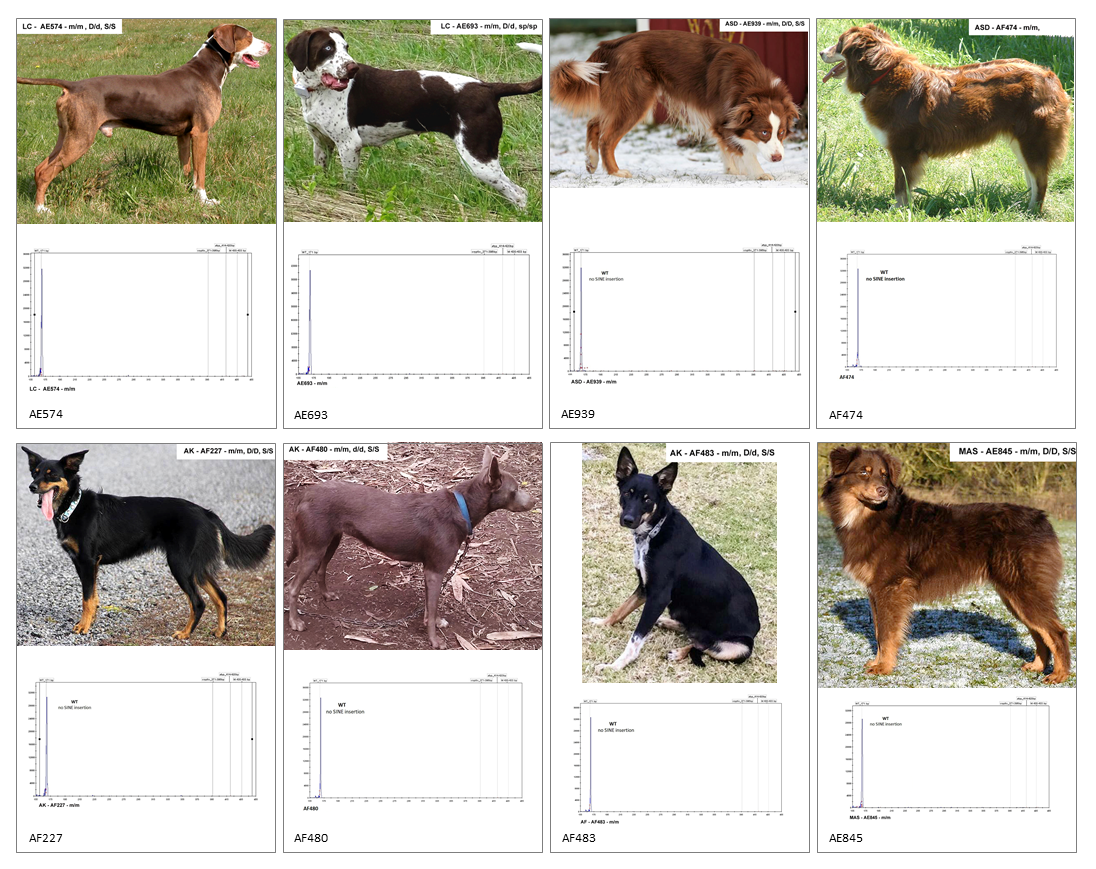

Supplement: S1 Fig — , subdivided into Figures A-X in S1 Fig according to the individual Merle allelic combinations, shows photographs and respective Merle allelic chromatograms of all 181 dogs included in our study. For detailed description of the major phenotypic features associated with the individual genotypes please refer to the main body of the article that can be found in Fig 3. The individual subdivisions of S1 Fig are the following: Figure A in S1 Fig Genotype/phenotype correlations: m/m; Figure B in S1 Fig Genotype/phenotype correlations: m/Mc; Figure C in S1 Fig Genotype/phenotype correlations: Mc/Mc; Figure D in S1 Fig Genotype / phenotype correlations: m/Mc+; Figure E in S1 Fig Genotype/phenotype correlations: Mc+/Mc+; Figure F in S1 Fig Genotype/phenotype correlations: m/Ma; Figure G in S1 Fig Genotype/phenotype correlations: Mc/Ma; Figure H in S1 Fig Genotype/phenotype correlations: Ma/Ma; Figure I in S1 Fig Genotype/phenotype correlations: m/Ma+; Figure J in S1 Fig Genotype/phenotype correlations: Mc/Ma+; Figure K in S1 Fig Genotype/phenotype correlations: Mc+/Ma+; Figure L in S1 Fig Genotype/phenotype correlations: Ma+/Ma+; Figure M in S1 Fig Genotype/phenotype correlations: m/M; Figure N in S1 Fig Genotype/phenotype correlations: Mc/M; Figure O in S1 Fig Genotype/phenotype correlations: Mc+/M; Figure P in S1 Fig Genotype/phenotype correlations: Ma/M; Figure Q in S1 Fig Genotype/phenotype correlations: Ma+/M; Figure R in S1 Fig Genotype/phenotype correlations: M/M; Figure S in S1 Fig Genotype/phenotype correlations: m/Mh; Figure T in S1 Fig Genotype/phenotype correlations: Mc/Mh; Figure U in S1 Fig Genotype/phenotype correlations: Mc+/Mh; Figure V in S1 Fig Genotype/phenotype correlations: Ma/Mh; Figure W in S1 Fig Genotype/phenotype correlations: M/Mh; Figure X in S1 Fig Genotype/phenotype correlations: Mh/Mh. (ZIP) [file pone.0198536.s007.zip › Figure A in S1 Fig_Langevin et al.tif]

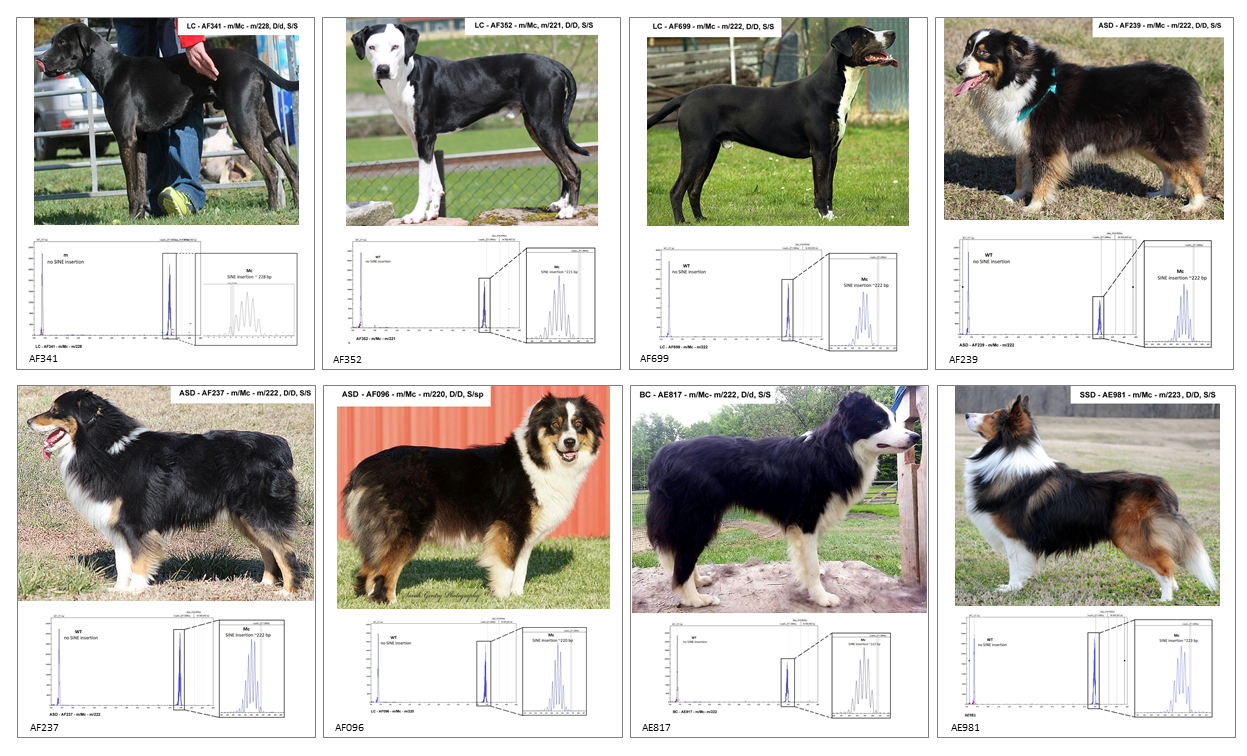

Supplement: S1 Fig — , subdivided into Figures A-X in S1 Fig according to the individual Merle allelic combinations, shows photographs and respective Merle allelic chromatograms of all 181 dogs included in our study. For detailed description of the major phenotypic features associated with the individual genotypes please refer to the main body of the article that can be found in Fig 3. The individual subdivisions of S1 Fig are the following: Figure A in S1 Fig Genotype/phenotype correlations: m/m; Figure B in S1 Fig Genotype/phenotype correlations: m/Mc; Figure C in S1 Fig Genotype/phenotype correlations: Mc/Mc; Figure D in S1 Fig Genotype / phenotype correlations: m/Mc+; Figure E in S1 Fig Genotype/phenotype correlations: Mc+/Mc+; Figure F in S1 Fig Genotype/phenotype correlations: m/Ma; Figure G in S1 Fig Genotype/phenotype correlations: Mc/Ma; Figure H in S1 Fig Genotype/phenotype correlations: Ma/Ma; Figure I in S1 Fig Genotype/phenotype correlations: m/Ma+; Figure J in S1 Fig Genotype/phenotype correlations: Mc/Ma+; Figure K in S1 Fig Genotype/phenotype correlations: Mc+/Ma+; Figure L in S1 Fig Genotype/phenotype correlations: Ma+/Ma+; Figure M in S1 Fig Genotype/phenotype correlations: m/M; Figure N in S1 Fig Genotype/phenotype correlations: Mc/M; Figure O in S1 Fig Genotype/phenotype correlations: Mc+/M; Figure P in S1 Fig Genotype/phenotype correlations: Ma/M; Figure Q in S1 Fig Genotype/phenotype correlations: Ma+/M; Figure R in S1 Fig Genotype/phenotype correlations: M/M; Figure S in S1 Fig Genotype/phenotype correlations: m/Mh; Figure T in S1 Fig Genotype/phenotype correlations: Mc/Mh; Figure U in S1 Fig Genotype/phenotype correlations: Mc+/Mh; Figure V in S1 Fig Genotype/phenotype correlations: Ma/Mh; Figure W in S1 Fig Genotype/phenotype correlations: M/Mh; Figure X in S1 Fig Genotype/phenotype correlations: Mh/Mh. (ZIP) [file pone.0198536.s007.zip › Figure B in S1 Fig_Langevin et al.tif]

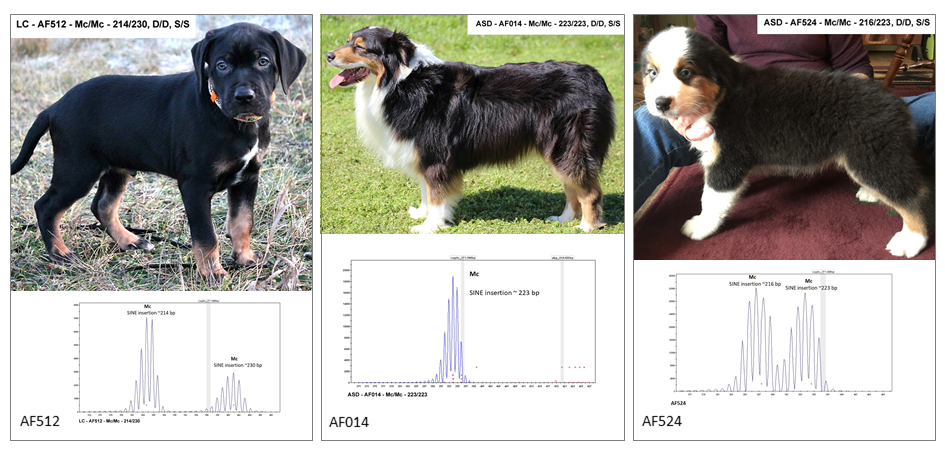

Supplement: S1 Fig — , subdivided into Figures A-X in S1 Fig according to the individual Merle allelic combinations, shows photographs and respective Merle allelic chromatograms of all 181 dogs included in our study. For detailed description of the major phenotypic features associated with the individual genotypes please refer to the main body of the article that can be found in Fig 3. The individual subdivisions of S1 Fig are the following: Figure A in S1 Fig Genotype/phenotype correlations: m/m; Figure B in S1 Fig Genotype/phenotype correlations: m/Mc; Figure C in S1 Fig Genotype/phenotype correlations: Mc/Mc; Figure D in S1 Fig Genotype / phenotype correlations: m/Mc+; Figure E in S1 Fig Genotype/phenotype correlations: Mc+/Mc+; Figure F in S1 Fig Genotype/phenotype correlations: m/Ma; Figure G in S1 Fig Genotype/phenotype correlations: Mc/Ma; Figure H in S1 Fig Genotype/phenotype correlations: Ma/Ma; Figure I in S1 Fig Genotype/phenotype correlations: m/Ma+; Figure J in S1 Fig Genotype/phenotype correlations: Mc/Ma+; Figure K in S1 Fig Genotype/phenotype correlations: Mc+/Ma+; Figure L in S1 Fig Genotype/phenotype correlations: Ma+/Ma+; Figure M in S1 Fig Genotype/phenotype correlations: m/M; Figure N in S1 Fig Genotype/phenotype correlations: Mc/M; Figure O in S1 Fig Genotype/phenotype correlations: Mc+/M; Figure P in S1 Fig Genotype/phenotype correlations: Ma/M; Figure Q in S1 Fig Genotype/phenotype correlations: Ma+/M; Figure R in S1 Fig Genotype/phenotype correlations: M/M; Figure S in S1 Fig Genotype/phenotype correlations: m/Mh; Figure T in S1 Fig Genotype/phenotype correlations: Mc/Mh; Figure U in S1 Fig Genotype/phenotype correlations: Mc+/Mh; Figure V in S1 Fig Genotype/phenotype correlations: Ma/Mh; Figure W in S1 Fig Genotype/phenotype correlations: M/Mh; Figure X in S1 Fig Genotype/phenotype correlations: Mh/Mh. (ZIP) [file pone.0198536.s007.zip › Figure C in S1 Fig_Langevin et al.tif]

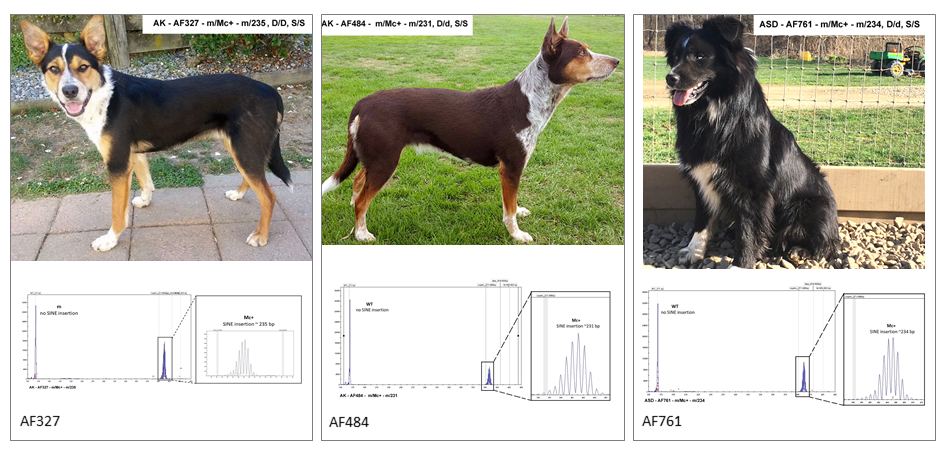

Supplement: S1 Fig — , subdivided into Figures A-X in S1 Fig according to the individual Merle allelic combinations, shows photographs and respective Merle allelic chromatograms of all 181 dogs included in our study. For detailed description of the major phenotypic features associated with the individual genotypes please refer to the main body of the article that can be found in Fig 3. The individual subdivisions of S1 Fig are the following: Figure A in S1 Fig Genotype/phenotype correlations: m/m; Figure B in S1 Fig Genotype/phenotype correlations: m/Mc; Figure C in S1 Fig Genotype/phenotype correlations: Mc/Mc; Figure D in S1 Fig Genotype / phenotype correlations: m/Mc+; Figure E in S1 Fig Genotype/phenotype correlations: Mc+/Mc+; Figure F in S1 Fig Genotype/phenotype correlations: m/Ma; Figure G in S1 Fig Genotype/phenotype correlations: Mc/Ma; Figure H in S1 Fig Genotype/phenotype correlations: Ma/Ma; Figure I in S1 Fig Genotype/phenotype correlations: m/Ma+; Figure J in S1 Fig Genotype/phenotype correlations: Mc/Ma+; Figure K in S1 Fig Genotype/phenotype correlations: Mc+/Ma+; Figure L in S1 Fig Genotype/phenotype correlations: Ma+/Ma+; Figure M in S1 Fig Genotype/phenotype correlations: m/M; Figure N in S1 Fig Genotype/phenotype correlations: Mc/M; Figure O in S1 Fig Genotype/phenotype correlations: Mc+/M; Figure P in S1 Fig Genotype/phenotype correlations: Ma/M; Figure Q in S1 Fig Genotype/phenotype correlations: Ma+/M; Figure R in S1 Fig Genotype/phenotype correlations: M/M; Figure S in S1 Fig Genotype/phenotype correlations: m/Mh; Figure T in S1 Fig Genotype/phenotype correlations: Mc/Mh; Figure U in S1 Fig Genotype/phenotype correlations: Mc+/Mh; Figure V in S1 Fig Genotype/phenotype correlations: Ma/Mh; Figure W in S1 Fig Genotype/phenotype correlations: M/Mh; Figure X in S1 Fig Genotype/phenotype correlations: Mh/Mh. (ZIP) [file pone.0198536.s007.zip › Figure D in S1 Fig_Langevin et al.tif]

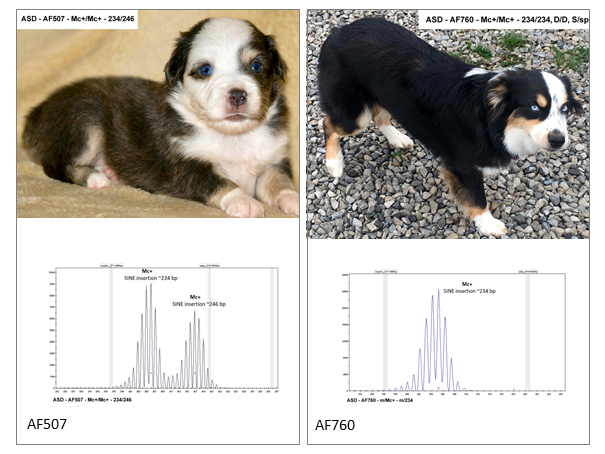

Supplement: S1 Fig — , subdivided into Figures A-X in S1 Fig according to the individual Merle allelic combinations, shows photographs and respective Merle allelic chromatograms of all 181 dogs included in our study. For detailed description of the major phenotypic features associated with the individual genotypes please refer to the main body of the article that can be found in Fig 3. The individual subdivisions of S1 Fig are the following: Figure A in S1 Fig Genotype/phenotype correlations: m/m; Figure B in S1 Fig Genotype/phenotype correlations: m/Mc; Figure C in S1 Fig Genotype/phenotype correlations: Mc/Mc; Figure D in S1 Fig Genotype / phenotype correlations: m/Mc+; Figure E in S1 Fig Genotype/phenotype correlations: Mc+/Mc+; Figure F in S1 Fig Genotype/phenotype correlations: m/Ma; Figure G in S1 Fig Genotype/phenotype correlations: Mc/Ma; Figure H in S1 Fig Genotype/phenotype correlations: Ma/Ma; Figure I in S1 Fig Genotype/phenotype correlations: m/Ma+; Figure J in S1 Fig Genotype/phenotype correlations: Mc/Ma+; Figure K in S1 Fig Genotype/phenotype correlations: Mc+/Ma+; Figure L in S1 Fig Genotype/phenotype correlations: Ma+/Ma+; Figure M in S1 Fig Genotype/phenotype correlations: m/M; Figure N in S1 Fig Genotype/phenotype correlations: Mc/M; Figure O in S1 Fig Genotype/phenotype correlations: Mc+/M; Figure P in S1 Fig Genotype/phenotype correlations: Ma/M; Figure Q in S1 Fig Genotype/phenotype correlations: Ma+/M; Figure R in S1 Fig Genotype/phenotype correlations: M/M; Figure S in S1 Fig Genotype/phenotype correlations: m/Mh; Figure T in S1 Fig Genotype/phenotype correlations: Mc/Mh; Figure U in S1 Fig Genotype/phenotype correlations: Mc+/Mh; Figure V in S1 Fig Genotype/phenotype correlations: Ma/Mh; Figure W in S1 Fig Genotype/phenotype correlations: M/Mh; Figure X in S1 Fig Genotype/phenotype correlations: Mh/Mh. (ZIP) [file pone.0198536.s007.zip › Figure E in S1 Fig_Langevin et al.tif]

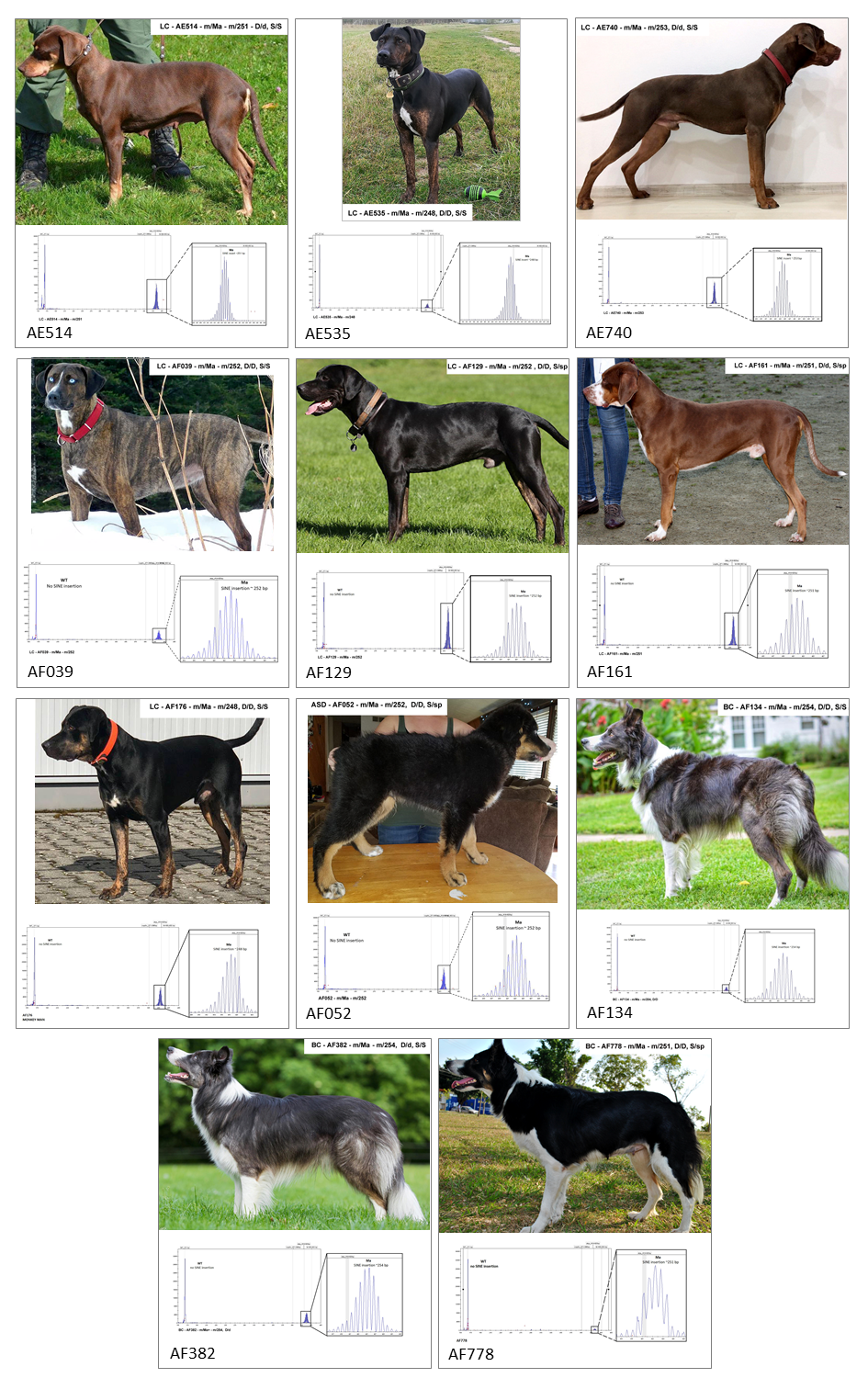

Supplement: S1 Fig — , subdivided into Figures A-X in S1 Fig according to the individual Merle allelic combinations, shows photographs and respective Merle allelic chromatograms of all 181 dogs included in our study. For detailed description of the major phenotypic features associated with the individual genotypes please refer to the main body of the article that can be found in Fig 3. The individual subdivisions of S1 Fig are the following: Figure A in S1 Fig Genotype/phenotype correlations: m/m; Figure B in S1 Fig Genotype/phenotype correlations: m/Mc; Figure C in S1 Fig Genotype/phenotype correlations: Mc/Mc; Figure D in S1 Fig Genotype / phenotype correlations: m/Mc+; Figure E in S1 Fig Genotype/phenotype correlations: Mc+/Mc+; Figure F in S1 Fig Genotype/phenotype correlations: m/Ma; Figure G in S1 Fig Genotype/phenotype correlations: Mc/Ma; Figure H in S1 Fig Genotype/phenotype correlations: Ma/Ma; Figure I in S1 Fig Genotype/phenotype correlations: m/Ma+; Figure J in S1 Fig Genotype/phenotype correlations: Mc/Ma+; Figure K in S1 Fig Genotype/phenotype correlations: Mc+/Ma+; Figure L in S1 Fig Genotype/phenotype correlations: Ma+/Ma+; Figure M in S1 Fig Genotype/phenotype correlations: m/M; Figure N in S1 Fig Genotype/phenotype correlations: Mc/M; Figure O in S1 Fig Genotype/phenotype correlations: Mc+/M; Figure P in S1 Fig Genotype/phenotype correlations: Ma/M; Figure Q in S1 Fig Genotype/phenotype correlations: Ma+/M; Figure R in S1 Fig Genotype/phenotype correlations: M/M; Figure S in S1 Fig Genotype/phenotype correlations: m/Mh; Figure T in S1 Fig Genotype/phenotype correlations: Mc/Mh; Figure U in S1 Fig Genotype/phenotype correlations: Mc+/Mh; Figure V in S1 Fig Genotype/phenotype correlations: Ma/Mh; Figure W in S1 Fig Genotype/phenotype correlations: M/Mh; Figure X in S1 Fig Genotype/phenotype correlations: Mh/Mh. (ZIP) [file pone.0198536.s007.zip › Figure F in S1 Fig_Langevin et al.tif]

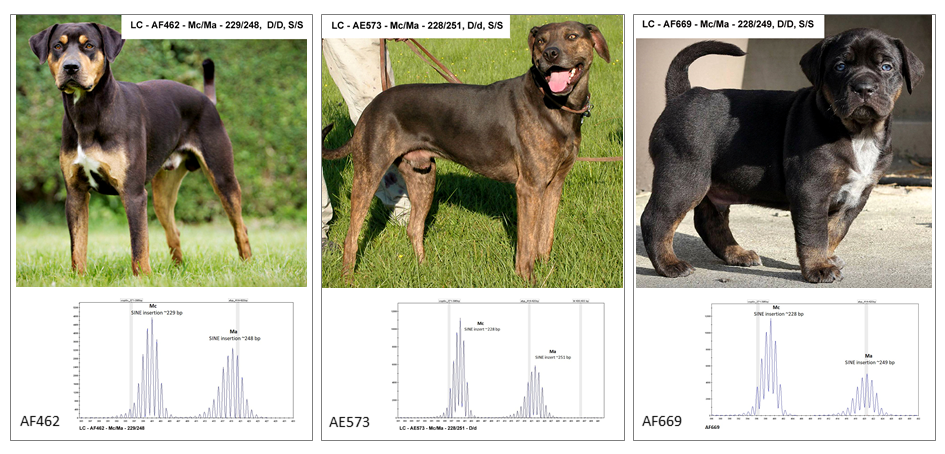

Supplement: S1 Fig — , subdivided into Figures A-X in S1 Fig according to the individual Merle allelic combinations, shows photographs and respective Merle allelic chromatograms of all 181 dogs included in our study. For detailed description of the major phenotypic features associated with the individual genotypes please refer to the main body of the article that can be found in Fig 3. The individual subdivisions of S1 Fig are the following: Figure A in S1 Fig Genotype/phenotype correlations: m/m; Figure B in S1 Fig Genotype/phenotype correlations: m/Mc; Figure C in S1 Fig Genotype/phenotype correlations: Mc/Mc; Figure D in S1 Fig Genotype / phenotype correlations: m/Mc+; Figure E in S1 Fig Genotype/phenotype correlations: Mc+/Mc+; Figure F in S1 Fig Genotype/phenotype correlations: m/Ma; Figure G in S1 Fig Genotype/phenotype correlations: Mc/Ma; Figure H in S1 Fig Genotype/phenotype correlations: Ma/Ma; Figure I in S1 Fig Genotype/phenotype correlations: m/Ma+; Figure J in S1 Fig Genotype/phenotype correlations: Mc/Ma+; Figure K in S1 Fig Genotype/phenotype correlations: Mc+/Ma+; Figure L in S1 Fig Genotype/phenotype correlations: Ma+/Ma+; Figure M in S1 Fig Genotype/phenotype correlations: m/M; Figure N in S1 Fig Genotype/phenotype correlations: Mc/M; Figure O in S1 Fig Genotype/phenotype correlations: Mc+/M; Figure P in S1 Fig Genotype/phenotype correlations: Ma/M; Figure Q in S1 Fig Genotype/phenotype correlations: Ma+/M; Figure R in S1 Fig Genotype/phenotype correlations: M/M; Figure S in S1 Fig Genotype/phenotype correlations: m/Mh; Figure T in S1 Fig Genotype/phenotype correlations: Mc/Mh; Figure U in S1 Fig Genotype/phenotype correlations: Mc+/Mh; Figure V in S1 Fig Genotype/phenotype correlations: Ma/Mh; Figure W in S1 Fig Genotype/phenotype correlations: M/Mh; Figure X in S1 Fig Genotype/phenotype correlations: Mh/Mh. (ZIP) [file pone.0198536.s007.zip › Figure G in S1 Fig_Langevin et al.tif]

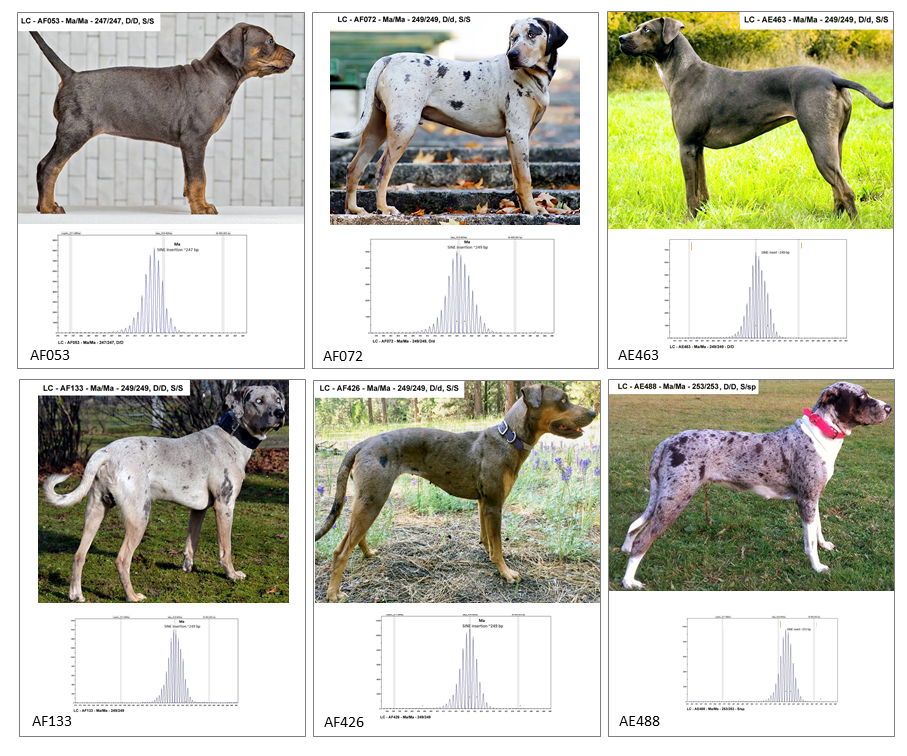

Supplement: S1 Fig — , subdivided into Figures A-X in S1 Fig according to the individual Merle allelic combinations, shows photographs and respective Merle allelic chromatograms of all 181 dogs included in our study. For detailed description of the major phenotypic features associated with the individual genotypes please refer to the main body of the article that can be found in Fig 3. The individual subdivisions of S1 Fig are the following: Figure A in S1 Fig Genotype/phenotype correlations: m/m; Figure B in S1 Fig Genotype/phenotype correlations: m/Mc; Figure C in S1 Fig Genotype/phenotype correlations: Mc/Mc; Figure D in S1 Fig Genotype / phenotype correlations: m/Mc+; Figure E in S1 Fig Genotype/phenotype correlations: Mc+/Mc+; Figure F in S1 Fig Genotype/phenotype correlations: m/Ma; Figure G in S1 Fig Genotype/phenotype correlations: Mc/Ma; Figure H in S1 Fig Genotype/phenotype correlations: Ma/Ma; Figure I in S1 Fig Genotype/phenotype correlations: m/Ma+; Figure J in S1 Fig Genotype/phenotype correlations: Mc/Ma+; Figure K in S1 Fig Genotype/phenotype correlations: Mc+/Ma+; Figure L in S1 Fig Genotype/phenotype correlations: Ma+/Ma+; Figure M in S1 Fig Genotype/phenotype correlations: m/M; Figure N in S1 Fig Genotype/phenotype correlations: Mc/M; Figure O in S1 Fig Genotype/phenotype correlations: Mc+/M; Figure P in S1 Fig Genotype/phenotype correlations: Ma/M; Figure Q in S1 Fig Genotype/phenotype correlations: Ma+/M; Figure R in S1 Fig Genotype/phenotype correlations: M/M; Figure S in S1 Fig Genotype/phenotype correlations: m/Mh; Figure T in S1 Fig Genotype/phenotype correlations: Mc/Mh; Figure U in S1 Fig Genotype/phenotype correlations: Mc+/Mh; Figure V in S1 Fig Genotype/phenotype correlations: Ma/Mh; Figure W in S1 Fig Genotype/phenotype correlations: M/Mh; Figure X in S1 Fig Genotype/phenotype correlations: Mh/Mh. (ZIP) [file pone.0198536.s007.zip › Figure H in S1 Fig_Langevin et al.tif]

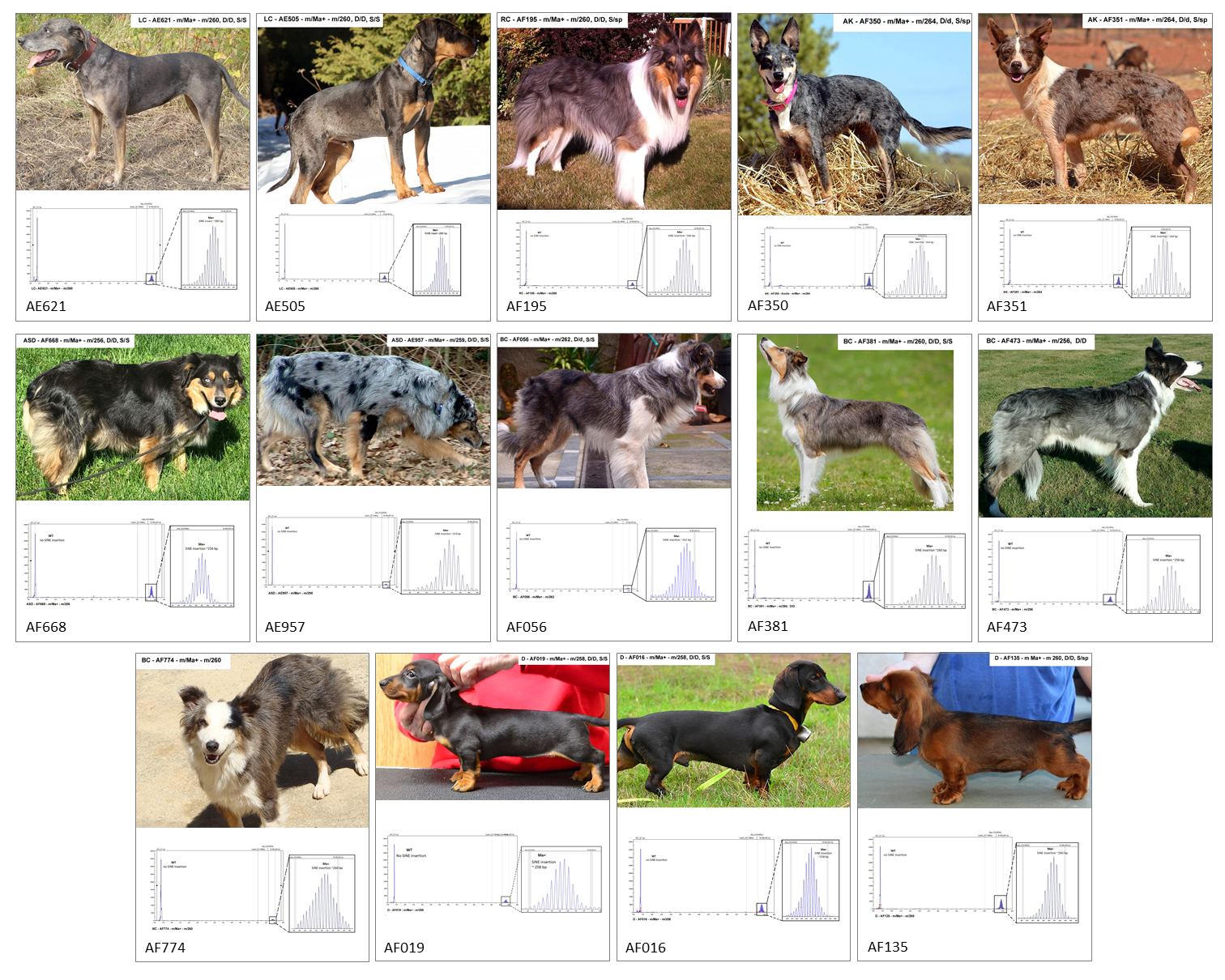

Supplement: S1 Fig — , subdivided into Figures A-X in S1 Fig according to the individual Merle allelic combinations, shows photographs and respective Merle allelic chromatograms of all 181 dogs included in our study. For detailed description of the major phenotypic features associated with the individual genotypes please refer to the main body of the article that can be found in Fig 3. The individual subdivisions of S1 Fig are the following: Figure A in S1 Fig Genotype/phenotype correlations: m/m; Figure B in S1 Fig Genotype/phenotype correlations: m/Mc; Figure C in S1 Fig Genotype/phenotype correlations: Mc/Mc; Figure D in S1 Fig Genotype / phenotype correlations: m/Mc+; Figure E in S1 Fig Genotype/phenotype correlations: Mc+/Mc+; Figure F in S1 Fig Genotype/phenotype correlations: m/Ma; Figure G in S1 Fig Genotype/phenotype correlations: Mc/Ma; Figure H in S1 Fig Genotype/phenotype correlations: Ma/Ma; Figure I in S1 Fig Genotype/phenotype correlations: m/Ma+; Figure J in S1 Fig Genotype/phenotype correlations: Mc/Ma+; Figure K in S1 Fig Genotype/phenotype correlations: Mc+/Ma+; Figure L in S1 Fig Genotype/phenotype correlations: Ma+/Ma+; Figure M in S1 Fig Genotype/phenotype correlations: m/M; Figure N in S1 Fig Genotype/phenotype correlations: Mc/M; Figure O in S1 Fig Genotype/phenotype correlations: Mc+/M; Figure P in S1 Fig Genotype/phenotype correlations: Ma/M; Figure Q in S1 Fig Genotype/phenotype correlations: Ma+/M; Figure R in S1 Fig Genotype/phenotype correlations: M/M; Figure S in S1 Fig Genotype/phenotype correlations: m/Mh; Figure T in S1 Fig Genotype/phenotype correlations: Mc/Mh; Figure U in S1 Fig Genotype/phenotype correlations: Mc+/Mh; Figure V in S1 Fig Genotype/phenotype correlations: Ma/Mh; Figure W in S1 Fig Genotype/phenotype correlations: M/Mh; Figure X in S1 Fig Genotype/phenotype correlations: Mh/Mh. (ZIP) [file pone.0198536.s007.zip › Figure I in S1 Fig_Langevin et al.tif]

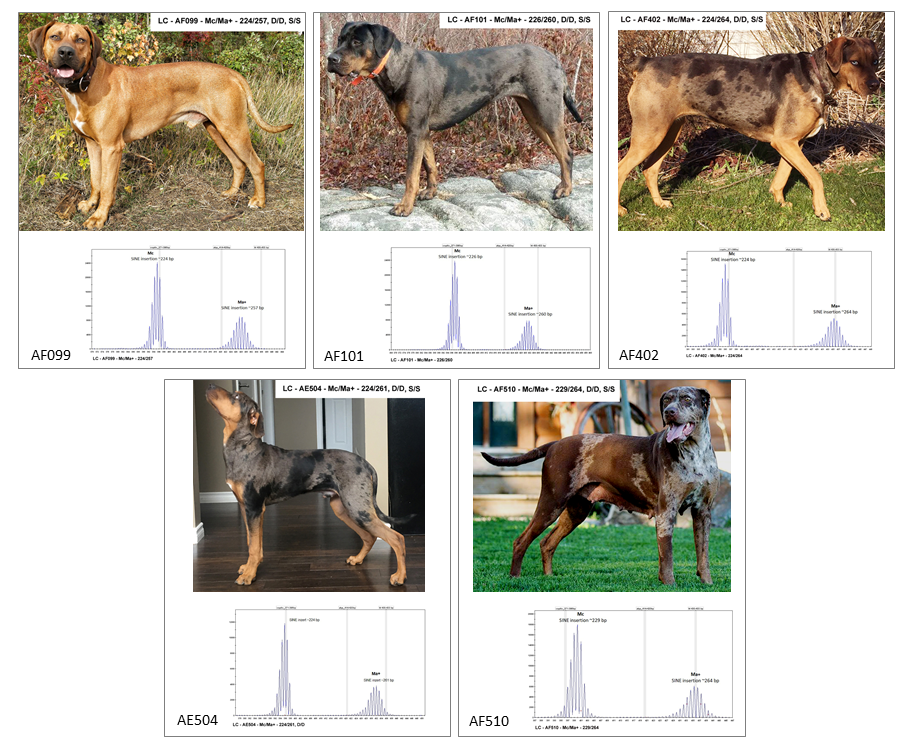

Supplement: S1 Fig — , subdivided into Figures A-X in S1 Fig according to the individual Merle allelic combinations, shows photographs and respective Merle allelic chromatograms of all 181 dogs included in our study. For detailed description of the major phenotypic features associated with the individual genotypes please refer to the main body of the article that can be found in Fig 3. The individual subdivisions of S1 Fig are the following: Figure A in S1 Fig Genotype/phenotype correlations: m/m; Figure B in S1 Fig Genotype/phenotype correlations: m/Mc; Figure C in S1 Fig Genotype/phenotype correlations: Mc/Mc; Figure D in S1 Fig Genotype / phenotype correlations: m/Mc+; Figure E in S1 Fig Genotype/phenotype correlations: Mc+/Mc+; Figure F in S1 Fig Genotype/phenotype correlations: m/Ma; Figure G in S1 Fig Genotype/phenotype correlations: Mc/Ma; Figure H in S1 Fig Genotype/phenotype correlations: Ma/Ma; Figure I in S1 Fig Genotype/phenotype correlations: m/Ma+; Figure J in S1 Fig Genotype/phenotype correlations: Mc/Ma+; Figure K in S1 Fig Genotype/phenotype correlations: Mc+/Ma+; Figure L in S1 Fig Genotype/phenotype correlations: Ma+/Ma+; Figure M in S1 Fig Genotype/phenotype correlations: m/M; Figure N in S1 Fig Genotype/phenotype correlations: Mc/M; Figure O in S1 Fig Genotype/phenotype correlations: Mc+/M; Figure P in S1 Fig Genotype/phenotype correlations: Ma/M; Figure Q in S1 Fig Genotype/phenotype correlations: Ma+/M; Figure R in S1 Fig Genotype/phenotype correlations: M/M; Figure S in S1 Fig Genotype/phenotype correlations: m/Mh; Figure T in S1 Fig Genotype/phenotype correlations: Mc/Mh; Figure U in S1 Fig Genotype/phenotype correlations: Mc+/Mh; Figure V in S1 Fig Genotype/phenotype correlations: Ma/Mh; Figure W in S1 Fig Genotype/phenotype correlations: M/Mh; Figure X in S1 Fig Genotype/phenotype correlations: Mh/Mh. (ZIP) [file pone.0198536.s007.zip › Figure J in S1 Fig_Langevin et al.tif]

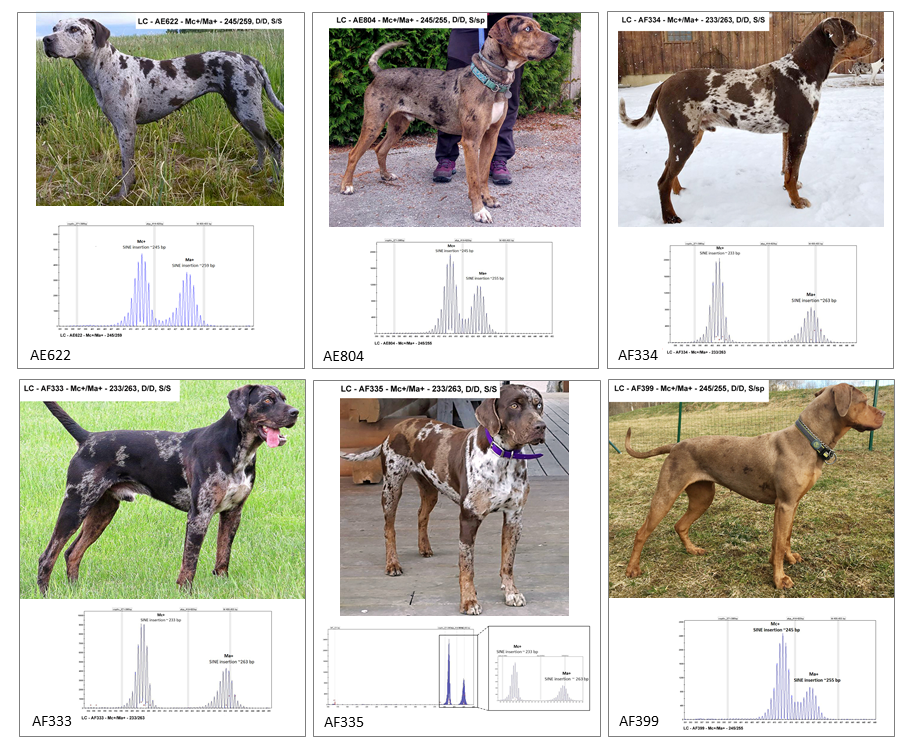

Supplement: S1 Fig — , subdivided into Figures A-X in S1 Fig according to the individual Merle allelic combinations, shows photographs and respective Merle allelic chromatograms of all 181 dogs included in our study. For detailed description of the major phenotypic features associated with the individual genotypes please refer to the main body of the article that can be found in Fig 3. The individual subdivisions of S1 Fig are the following: Figure A in S1 Fig Genotype/phenotype correlations: m/m; Figure B in S1 Fig Genotype/phenotype correlations: m/Mc; Figure C in S1 Fig Genotype/phenotype correlations: Mc/Mc; Figure D in S1 Fig Genotype / phenotype correlations: m/Mc+; Figure E in S1 Fig Genotype/phenotype correlations: Mc+/Mc+; Figure F in S1 Fig Genotype/phenotype correlations: m/Ma; Figure G in S1 Fig Genotype/phenotype correlations: Mc/Ma; Figure H in S1 Fig Genotype/phenotype correlations: Ma/Ma; Figure I in S1 Fig Genotype/phenotype correlations: m/Ma+; Figure J in S1 Fig Genotype/phenotype correlations: Mc/Ma+; Figure K in S1 Fig Genotype/phenotype correlations: Mc+/Ma+; Figure L in S1 Fig Genotype/phenotype correlations: Ma+/Ma+; Figure M in S1 Fig Genotype/phenotype correlations: m/M; Figure N in S1 Fig Genotype/phenotype correlations: Mc/M; Figure O in S1 Fig Genotype/phenotype correlations: Mc+/M; Figure P in S1 Fig Genotype/phenotype correlations: Ma/M; Figure Q in S1 Fig Genotype/phenotype correlations: Ma+/M; Figure R in S1 Fig Genotype/phenotype correlations: M/M; Figure S in S1 Fig Genotype/phenotype correlations: m/Mh; Figure T in S1 Fig Genotype/phenotype correlations: Mc/Mh; Figure U in S1 Fig Genotype/phenotype correlations: Mc+/Mh; Figure V in S1 Fig Genotype/phenotype correlations: Ma/Mh; Figure W in S1 Fig Genotype/phenotype correlations: M/Mh; Figure X in S1 Fig Genotype/phenotype correlations: Mh/Mh. (ZIP) [file pone.0198536.s007.zip › Figure K in S1 Fig_Langevin et al.tif]

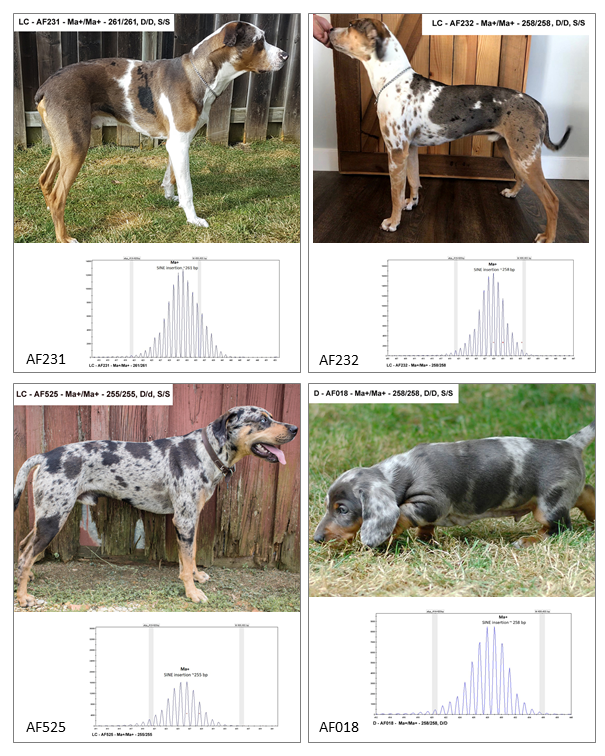

Supplement: S1 Fig — , subdivided into Figures A-X in S1 Fig according to the individual Merle allelic combinations, shows photographs and respective Merle allelic chromatograms of all 181 dogs included in our study. For detailed description of the major phenotypic features associated with the individual genotypes please refer to the main body of the article that can be found in Fig 3. The individual subdivisions of S1 Fig are the following: Figure A in S1 Fig Genotype/phenotype correlations: m/m; Figure B in S1 Fig Genotype/phenotype correlations: m/Mc; Figure C in S1 Fig Genotype/phenotype correlations: Mc/Mc; Figure D in S1 Fig Genotype / phenotype correlations: m/Mc+; Figure E in S1 Fig Genotype/phenotype correlations: Mc+/Mc+; Figure F in S1 Fig Genotype/phenotype correlations: m/Ma; Figure G in S1 Fig Genotype/phenotype correlations: Mc/Ma; Figure H in S1 Fig Genotype/phenotype correlations: Ma/Ma; Figure I in S1 Fig Genotype/phenotype correlations: m/Ma+; Figure J in S1 Fig Genotype/phenotype correlations: Mc/Ma+; Figure K in S1 Fig Genotype/phenotype correlations: Mc+/Ma+; Figure L in S1 Fig Genotype/phenotype correlations: Ma+/Ma+; Figure M in S1 Fig Genotype/phenotype correlations: m/M; Figure N in S1 Fig Genotype/phenotype correlations: Mc/M; Figure O in S1 Fig Genotype/phenotype correlations: Mc+/M; Figure P in S1 Fig Genotype/phenotype correlations: Ma/M; Figure Q in S1 Fig Genotype/phenotype correlations: Ma+/M; Figure R in S1 Fig Genotype/phenotype correlations: M/M; Figure S in S1 Fig Genotype/phenotype correlations: m/Mh; Figure T in S1 Fig Genotype/phenotype correlations: Mc/Mh; Figure U in S1 Fig Genotype/phenotype correlations: Mc+/Mh; Figure V in S1 Fig Genotype/phenotype correlations: Ma/Mh; Figure W in S1 Fig Genotype/phenotype correlations: M/Mh; Figure X in S1 Fig Genotype/phenotype correlations: Mh/Mh. (ZIP) [file pone.0198536.s007.zip › Figure L in S1 Fig_Langevin et al.tif]

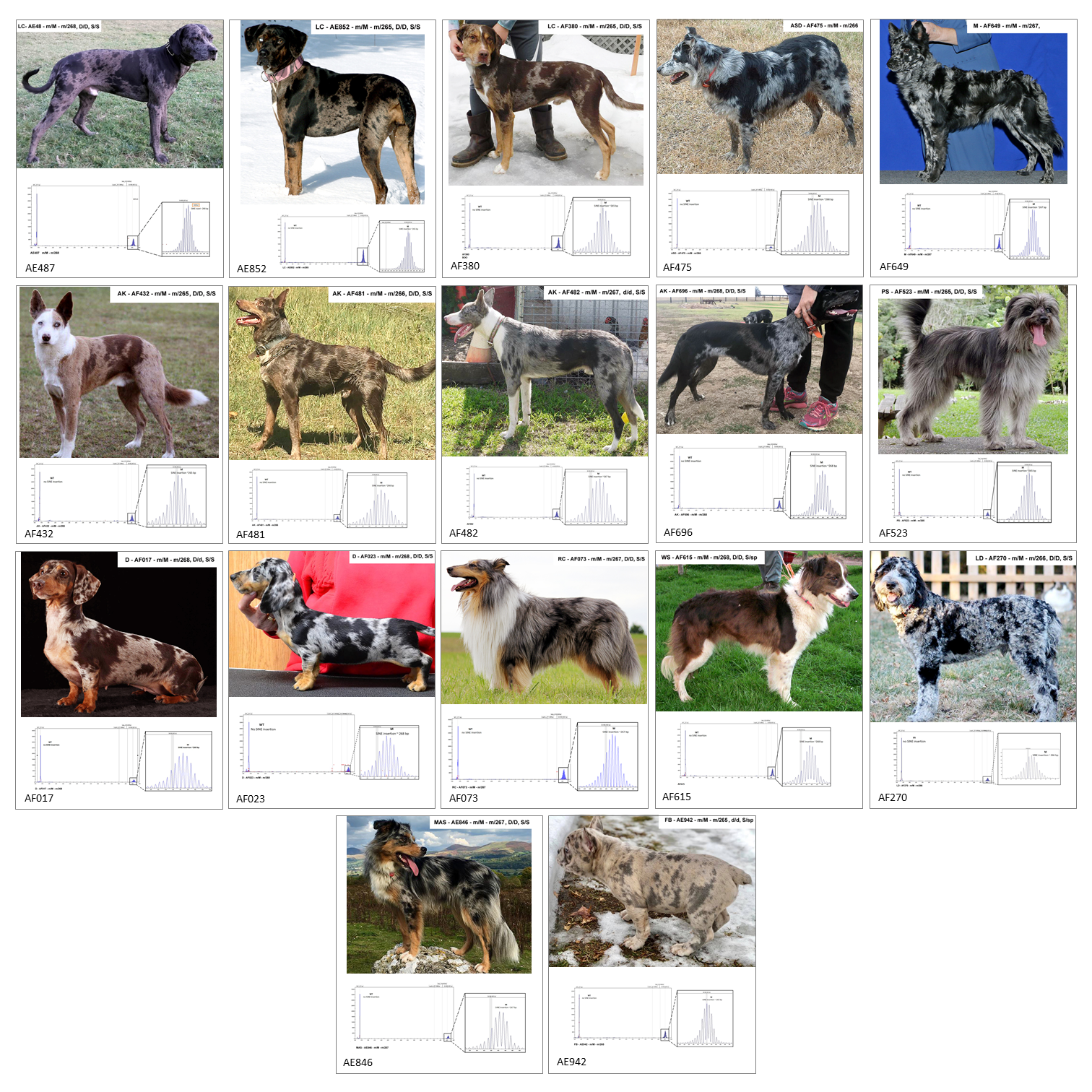

Supplement: S1 Fig — , subdivided into Figures A-X in S1 Fig according to the individual Merle allelic combinations, shows photographs and respective Merle allelic chromatograms of all 181 dogs included in our study. For detailed description of the major phenotypic features associated with the individual genotypes please refer to the main body of the article that can be found in Fig 3. The individual subdivisions of S1 Fig are the following: Figure A in S1 Fig Genotype/phenotype correlations: m/m; Figure B in S1 Fig Genotype/phenotype correlations: m/Mc; Figure C in S1 Fig Genotype/phenotype correlations: Mc/Mc; Figure D in S1 Fig Genotype / phenotype correlations: m/Mc+; Figure E in S1 Fig Genotype/phenotype correlations: Mc+/Mc+; Figure F in S1 Fig Genotype/phenotype correlations: m/Ma; Figure G in S1 Fig Genotype/phenotype correlations: Mc/Ma; Figure H in S1 Fig Genotype/phenotype correlations: Ma/Ma; Figure I in S1 Fig Genotype/phenotype correlations: m/Ma+; Figure J in S1 Fig Genotype/phenotype correlations: Mc/Ma+; Figure K in S1 Fig Genotype/phenotype correlations: Mc+/Ma+; Figure L in S1 Fig Genotype/phenotype correlations: Ma+/Ma+; Figure M in S1 Fig Genotype/phenotype correlations: m/M; Figure N in S1 Fig Genotype/phenotype correlations: Mc/M; Figure O in S1 Fig Genotype/phenotype correlations: Mc+/M; Figure P in S1 Fig Genotype/phenotype correlations: Ma/M; Figure Q in S1 Fig Genotype/phenotype correlations: Ma+/M; Figure R in S1 Fig Genotype/phenotype correlations: M/M; Figure S in S1 Fig Genotype/phenotype correlations: m/Mh; Figure T in S1 Fig Genotype/phenotype correlations: Mc/Mh; Figure U in S1 Fig Genotype/phenotype correlations: Mc+/Mh; Figure V in S1 Fig Genotype/phenotype correlations: Ma/Mh; Figure W in S1 Fig Genotype/phenotype correlations: M/Mh; Figure X in S1 Fig Genotype/phenotype correlations: Mh/Mh. (ZIP) [file pone.0198536.s007.zip › Figure M in S1 Fig_Langevin et al.tif]

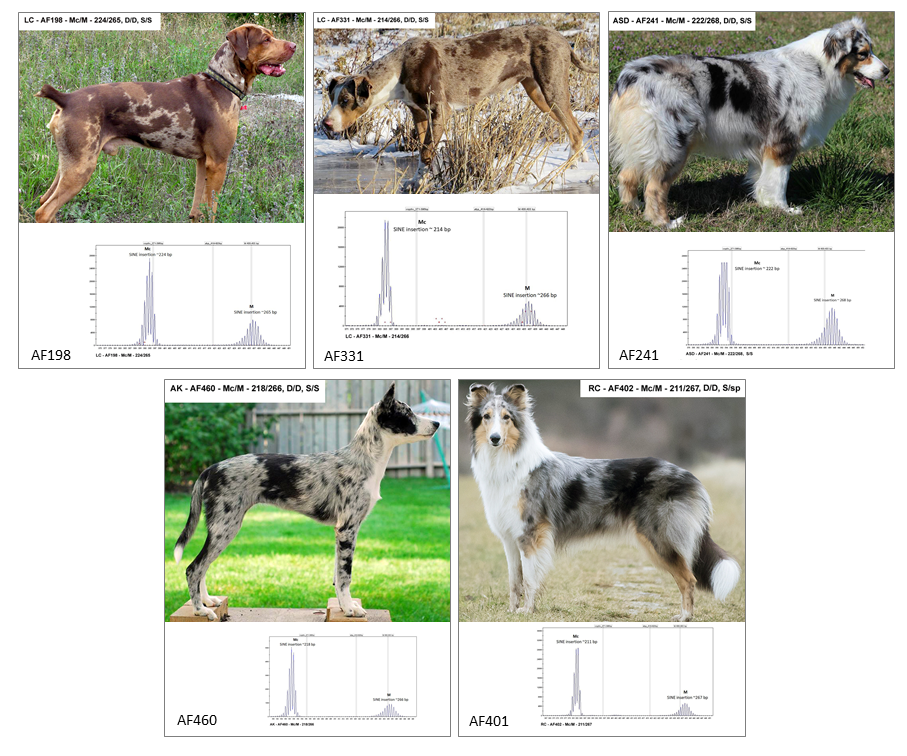

Supplement: S1 Fig — , subdivided into Figures A-X in S1 Fig according to the individual Merle allelic combinations, shows photographs and respective Merle allelic chromatograms of all 181 dogs included in our study. For detailed description of the major phenotypic features associated with the individual genotypes please refer to the main body of the article that can be found in Fig 3. The individual subdivisions of S1 Fig are the following: Figure A in S1 Fig Genotype/phenotype correlations: m/m; Figure B in S1 Fig Genotype/phenotype correlations: m/Mc; Figure C in S1 Fig Genotype/phenotype correlations: Mc/Mc; Figure D in S1 Fig Genotype / phenotype correlations: m/Mc+; Figure E in S1 Fig Genotype/phenotype correlations: Mc+/Mc+; Figure F in S1 Fig Genotype/phenotype correlations: m/Ma; Figure G in S1 Fig Genotype/phenotype correlations: Mc/Ma; Figure H in S1 Fig Genotype/phenotype correlations: Ma/Ma; Figure I in S1 Fig Genotype/phenotype correlations: m/Ma+; Figure J in S1 Fig Genotype/phenotype correlations: Mc/Ma+; Figure K in S1 Fig Genotype/phenotype correlations: Mc+/Ma+; Figure L in S1 Fig Genotype/phenotype correlations: Ma+/Ma+; Figure M in S1 Fig Genotype/phenotype correlations: m/M; Figure N in S1 Fig Genotype/phenotype correlations: Mc/M; Figure O in S1 Fig Genotype/phenotype correlations: Mc+/M; Figure P in S1 Fig Genotype/phenotype correlations: Ma/M; Figure Q in S1 Fig Genotype/phenotype correlations: Ma+/M; Figure R in S1 Fig Genotype/phenotype correlations: M/M; Figure S in S1 Fig Genotype/phenotype correlations: m/Mh; Figure T in S1 Fig Genotype/phenotype correlations: Mc/Mh; Figure U in S1 Fig Genotype/phenotype correlations: Mc+/Mh; Figure V in S1 Fig Genotype/phenotype correlations: Ma/Mh; Figure W in S1 Fig Genotype/phenotype correlations: M/Mh; Figure X in S1 Fig Genotype/phenotype correlations: Mh/Mh. (ZIP) [file pone.0198536.s007.zip › Figure N in S1 Fig_Langevin et al.tif]

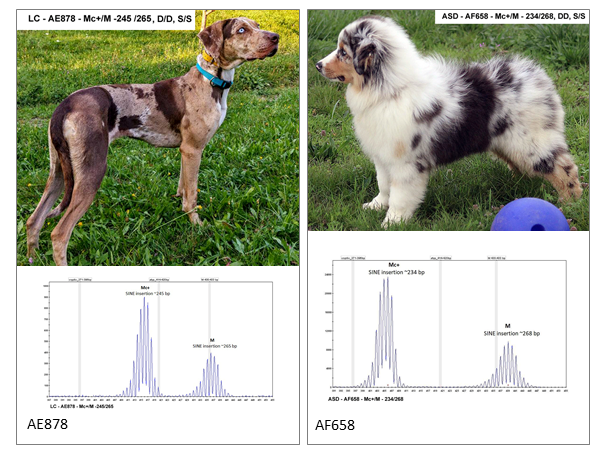

Supplement: S1 Fig — , subdivided into Figures A-X in S1 Fig according to the individual Merle allelic combinations, shows photographs and respective Merle allelic chromatograms of all 181 dogs included in our study. For detailed description of the major phenotypic features associated with the individual genotypes please refer to the main body of the article that can be found in Fig 3. The individual subdivisions of S1 Fig are the following: Figure A in S1 Fig Genotype/phenotype correlations: m/m; Figure B in S1 Fig Genotype/phenotype correlations: m/Mc; Figure C in S1 Fig Genotype/phenotype correlations: Mc/Mc; Figure D in S1 Fig Genotype / phenotype correlations: m/Mc+; Figure E in S1 Fig Genotype/phenotype correlations: Mc+/Mc+; Figure F in S1 Fig Genotype/phenotype correlations: m/Ma; Figure G in S1 Fig Genotype/phenotype correlations: Mc/Ma; Figure H in S1 Fig Genotype/phenotype correlations: Ma/Ma; Figure I in S1 Fig Genotype/phenotype correlations: m/Ma+; Figure J in S1 Fig Genotype/phenotype correlations: Mc/Ma+; Figure K in S1 Fig Genotype/phenotype correlations: Mc+/Ma+; Figure L in S1 Fig Genotype/phenotype correlations: Ma+/Ma+; Figure M in S1 Fig Genotype/phenotype correlations: m/M; Figure N in S1 Fig Genotype/phenotype correlations: Mc/M; Figure O in S1 Fig Genotype/phenotype correlations: Mc+/M; Figure P in S1 Fig Genotype/phenotype correlations: Ma/M; Figure Q in S1 Fig Genotype/phenotype correlations: Ma+/M; Figure R in S1 Fig Genotype/phenotype correlations: M/M; Figure S in S1 Fig Genotype/phenotype correlations: m/Mh; Figure T in S1 Fig Genotype/phenotype correlations: Mc/Mh; Figure U in S1 Fig Genotype/phenotype correlations: Mc+/Mh; Figure V in S1 Fig Genotype/phenotype correlations: Ma/Mh; Figure W in S1 Fig Genotype/phenotype correlations: M/Mh; Figure X in S1 Fig Genotype/phenotype correlations: Mh/Mh. (ZIP) [file pone.0198536.s007.zip › Figure O in S1 Fig_Langevin et al.tif]

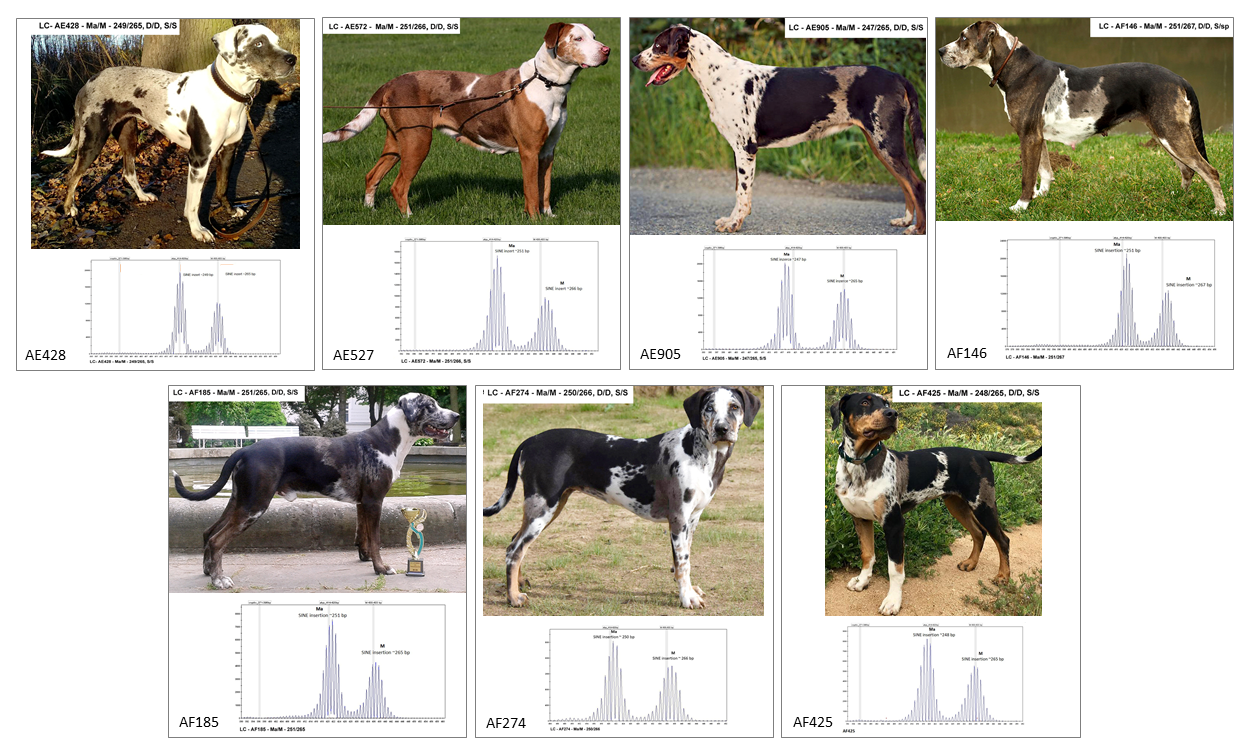

Supplement: S1 Fig — , subdivided into Figures A-X in S1 Fig according to the individual Merle allelic combinations, shows photographs and respective Merle allelic chromatograms of all 181 dogs included in our study. For detailed description of the major phenotypic features associated with the individual genotypes please refer to the main body of the article that can be found in Fig 3. The individual subdivisions of S1 Fig are the following: Figure A in S1 Fig Genotype/phenotype correlations: m/m; Figure B in S1 Fig Genotype/phenotype correlations: m/Mc; Figure C in S1 Fig Genotype/phenotype correlations: Mc/Mc; Figure D in S1 Fig Genotype / phenotype correlations: m/Mc+; Figure E in S1 Fig Genotype/phenotype correlations: Mc+/Mc+; Figure F in S1 Fig Genotype/phenotype correlations: m/Ma; Figure G in S1 Fig Genotype/phenotype correlations: Mc/Ma; Figure H in S1 Fig Genotype/phenotype correlations: Ma/Ma; Figure I in S1 Fig Genotype/phenotype correlations: m/Ma+; Figure J in S1 Fig Genotype/phenotype correlations: Mc/Ma+; Figure K in S1 Fig Genotype/phenotype correlations: Mc+/Ma+; Figure L in S1 Fig Genotype/phenotype correlations: Ma+/Ma+; Figure M in S1 Fig Genotype/phenotype correlations: m/M; Figure N in S1 Fig Genotype/phenotype correlations: Mc/M; Figure O in S1 Fig Genotype/phenotype correlations: Mc+/M; Figure P in S1 Fig Genotype/phenotype correlations: Ma/M; Figure Q in S1 Fig Genotype/phenotype correlations: Ma+/M; Figure R in S1 Fig Genotype/phenotype correlations: M/M; Figure S in S1 Fig Genotype/phenotype correlations: m/Mh; Figure T in S1 Fig Genotype/phenotype correlations: Mc/Mh; Figure U in S1 Fig Genotype/phenotype correlations: Mc+/Mh; Figure V in S1 Fig Genotype/phenotype correlations: Ma/Mh; Figure W in S1 Fig Genotype/phenotype correlations: M/Mh; Figure X in S1 Fig Genotype/phenotype correlations: Mh/Mh. (ZIP) [file pone.0198536.s007.zip › Figure P in S1 Fig_Langevin et al.tif]

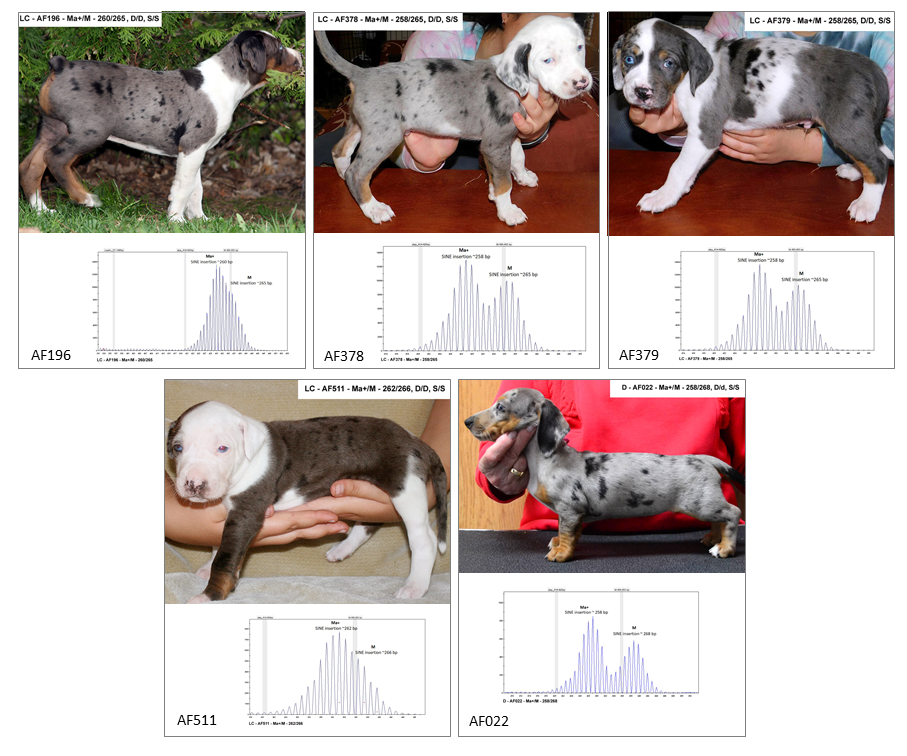

Supplement: S1 Fig — , subdivided into Figures A-X in S1 Fig according to the individual Merle allelic combinations, shows photographs and respective Merle allelic chromatograms of all 181 dogs included in our study. For detailed description of the major phenotypic features associated with the individual genotypes please refer to the main body of the article that can be found in Fig 3. The individual subdivisions of S1 Fig are the following: Figure A in S1 Fig Genotype/phenotype correlations: m/m; Figure B in S1 Fig Genotype/phenotype correlations: m/Mc; Figure C in S1 Fig Genotype/phenotype correlations: Mc/Mc; Figure D in S1 Fig Genotype / phenotype correlations: m/Mc+; Figure E in S1 Fig Genotype/phenotype correlations: Mc+/Mc+; Figure F in S1 Fig Genotype/phenotype correlations: m/Ma; Figure G in S1 Fig Genotype/phenotype correlations: Mc/Ma; Figure H in S1 Fig Genotype/phenotype correlations: Ma/Ma; Figure I in S1 Fig Genotype/phenotype correlations: m/Ma+; Figure J in S1 Fig Genotype/phenotype correlations: Mc/Ma+; Figure K in S1 Fig Genotype/phenotype correlations: Mc+/Ma+; Figure L in S1 Fig Genotype/phenotype correlations: Ma+/Ma+; Figure M in S1 Fig Genotype/phenotype correlations: m/M; Figure N in S1 Fig Genotype/phenotype correlations: Mc/M; Figure O in S1 Fig Genotype/phenotype correlations: Mc+/M; Figure P in S1 Fig Genotype/phenotype correlations: Ma/M; Figure Q in S1 Fig Genotype/phenotype correlations: Ma+/M; Figure R in S1 Fig Genotype/phenotype correlations: M/M; Figure S in S1 Fig Genotype/phenotype correlations: m/Mh; Figure T in S1 Fig Genotype/phenotype correlations: Mc/Mh; Figure U in S1 Fig Genotype/phenotype correlations: Mc+/Mh; Figure V in S1 Fig Genotype/phenotype correlations: Ma/Mh; Figure W in S1 Fig Genotype/phenotype correlations: M/Mh; Figure X in S1 Fig Genotype/phenotype correlations: Mh/Mh. (ZIP) [file pone.0198536.s007.zip › Figure Q in S1 Fig_Langevin et al.tif]

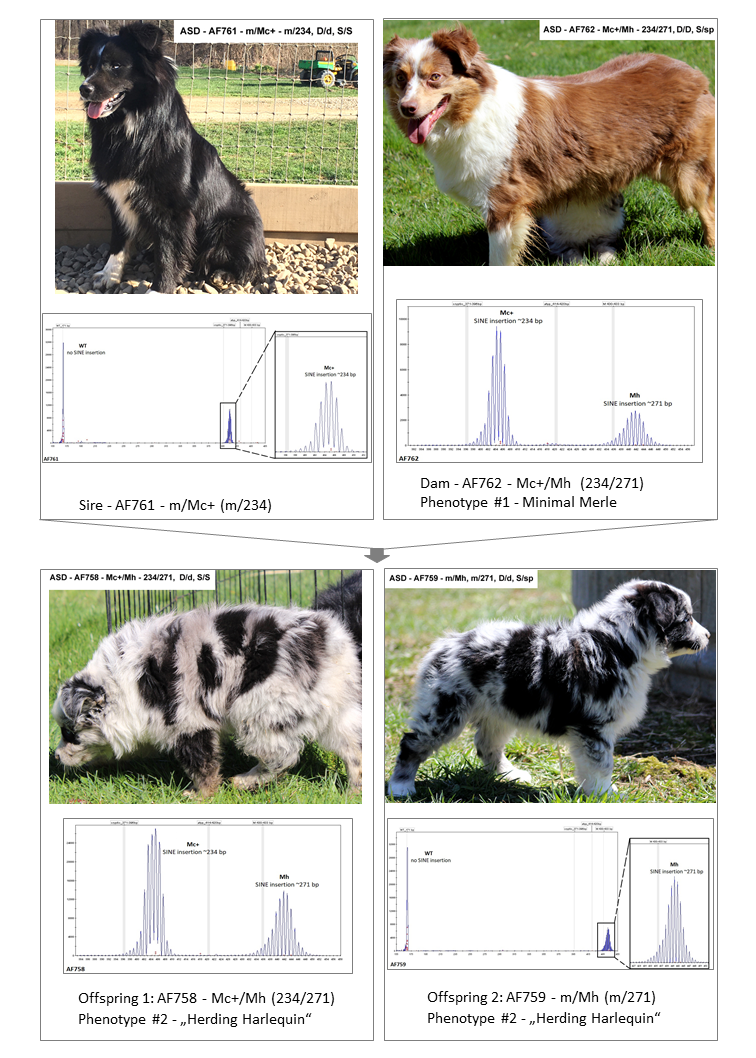

Supplement: S2 Fig — The dam–AF762 –is distinguished by a Mh phenotype as described in #1 - “Minimal Merle”. The two offspring–AF758 and AF759 both have a Mh phenotype as described in #2—The more classic pattern that is often referred to as “Herding Harlequin“. Both dam and offspring have the same length of Mh—271 bp. (TIF) [file pone.0198536.s008.tif]

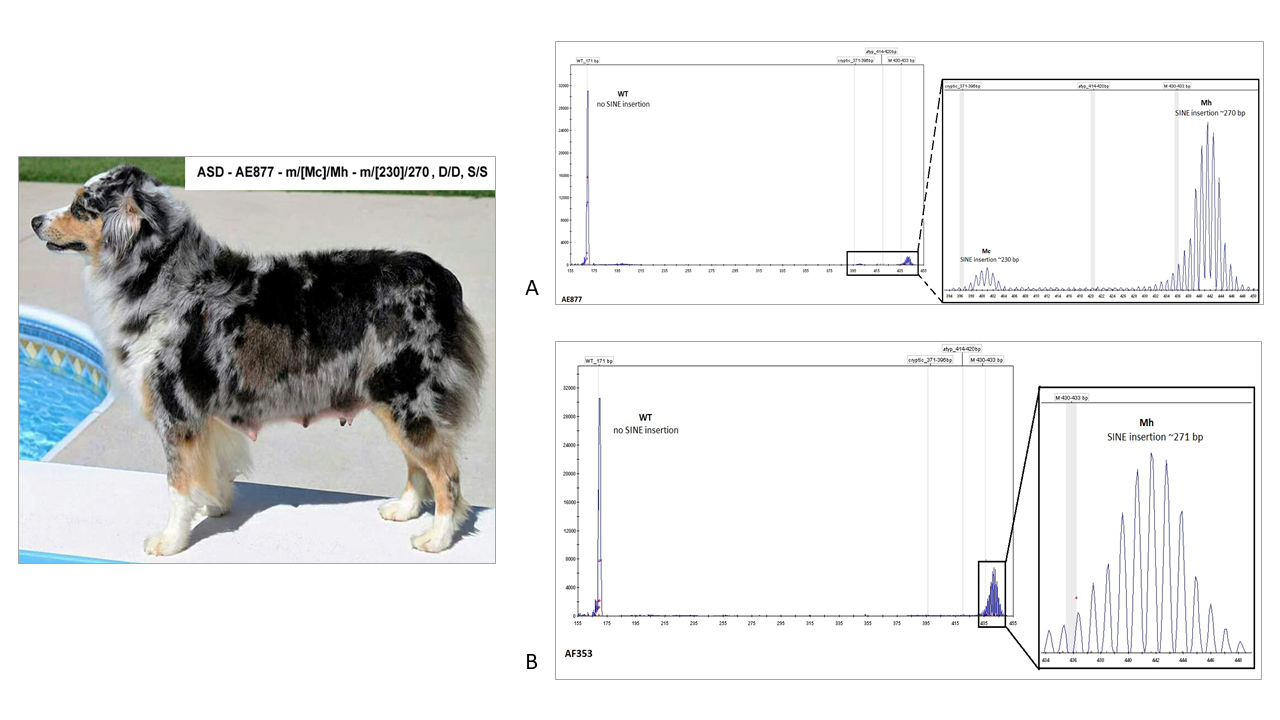

Supplement: S3 Fig — Difference in Merle allelic status between biological materials tested, proband AE877, a female ASD. (TIF) [file pone.0198536.s009.tif]

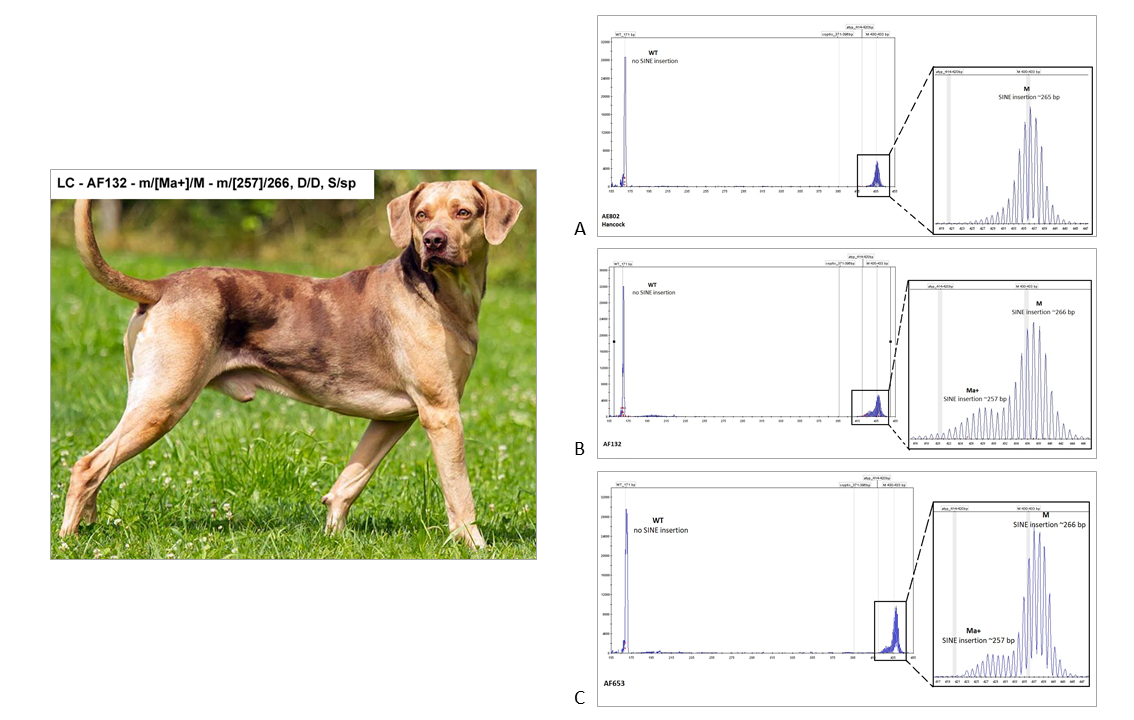

Supplement: S4 Fig — Difference in Merle allelic status among biological materials tested, proband AF132, a male LC. (TIF) [file pone.0198536.s010.tif]

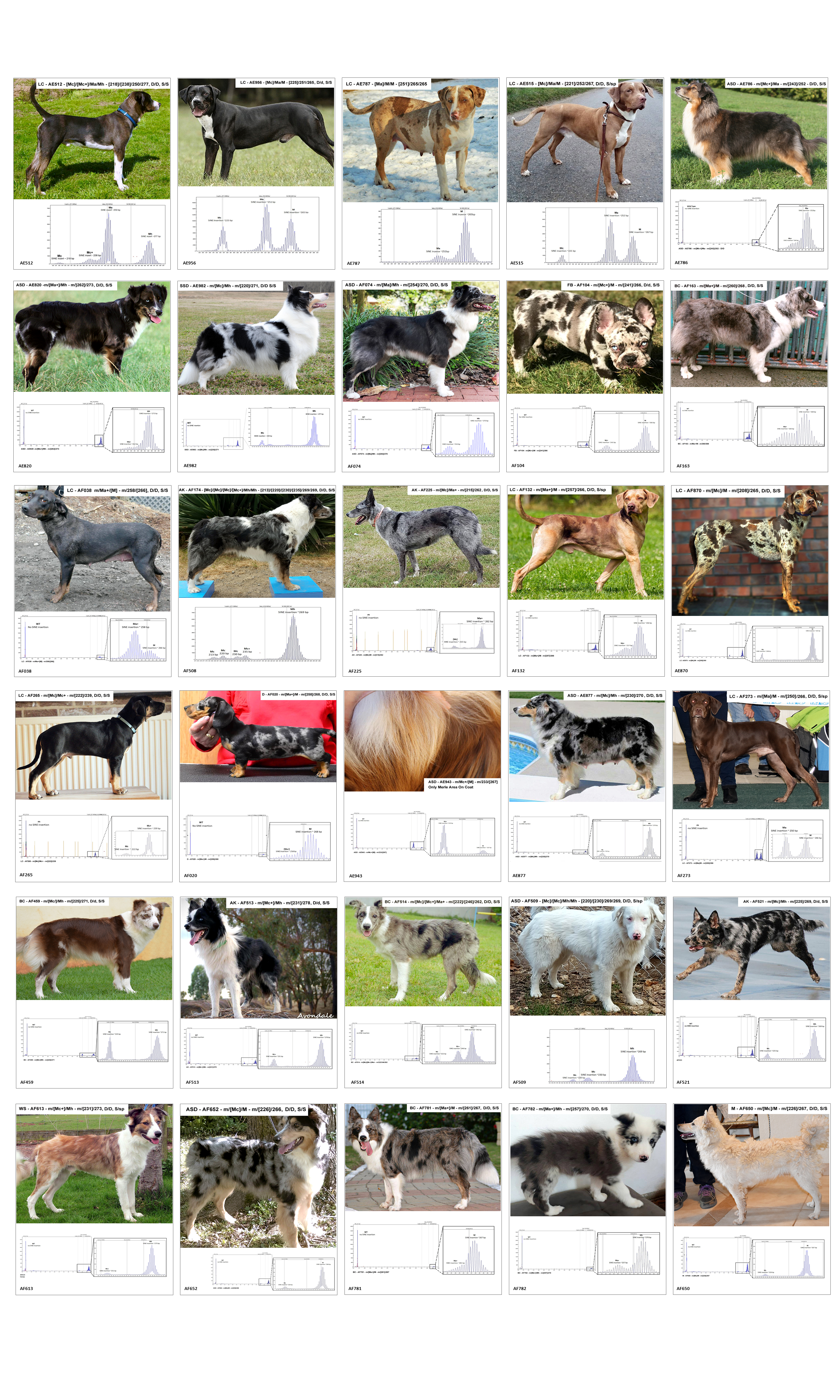

Supplement: S5 Fig — shows photographs and Merle chromatograms of the individuals given in S2 Table. (TIF) [file pone.0198536.s011.tif]

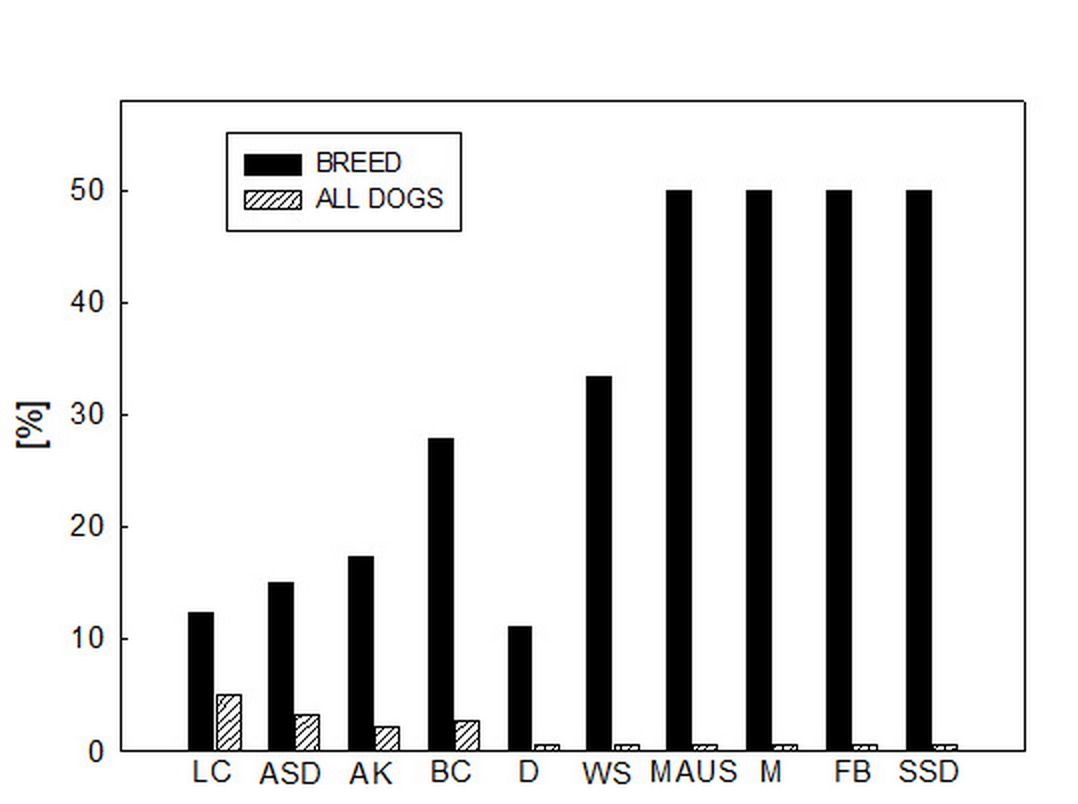

Supplement: S6 Fig — The individual values represent the percentage of total number of dogs in each breed with Merle mosaicism detected [ASD–Australian Shepherd Dog (n = 40), AK–Australian Koolie (n = 23), BC–Border Collie (n = 18), D–Dachshund (n = 9), LC–Louisiana Catahoula (n = 73), MAUS–Miniature Australian Shepherd (n = 2), SSD–Shetland Sheepdog (n = 2), FB–French Bulldog (n = 2), WS–Welsh Sheepdog (n = 3), M–Mudi (n = 2)] of all tested dogs (n = 181). (TIF) [file pone.0198536.s012.tif]

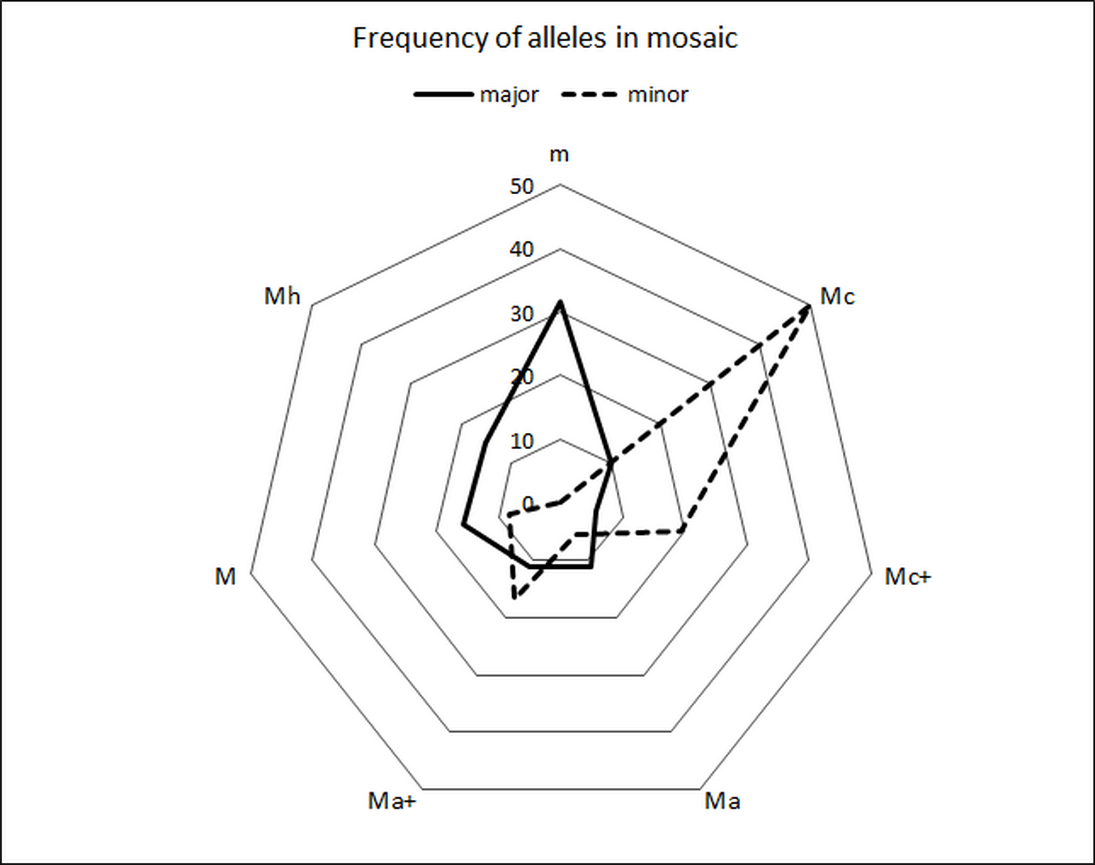

Supplement: S7 Fig — Relative frequency of individual alleles has been given as percentage of all alleles found as major or minor ones in mosaic dogs, calculated for all breeds together. (TIF) [file pone.0198536.s013.tif]
